# CD169^+^ Macrophage‐Targeted Immunomodulator to Restore Phagocytic Function and Enhance Antigen Presentation for Lymphatic Metastasis Eradication

**DOI:** 10.1002/advs.202514386

**Published:** 2025-11-16

**Authors:** Xiayun Chen, Lichong Lu, Yibin Liu, Ziqi Liang, Jianqiao Li, Zhouchuan Shao, Youzhi Tang, Jianhua Zou, Shiying Li, Xiaoyuan Chen

**Affiliations:** ^1^ The Guangdong Provincial Key Laboratory of Molecular Target & Clinical Pharmacology the NMPA and State Key Laboratory of Respiratory Disease the School of Pharmaceutical Sciences Guangzhou Medical University Guangzhou 511436 P. R. China; ^2^ Guangdong Provincial Key Laboratory of Veterinary Pharmaceutics Development and Safety Evaluation College of Veterinary Medicine South China Agricultural University Guangzhou 510642 P. R. China; ^3^ Department of Diagnostic Radiology Yong Loo Lin School of Medicine National University of Singapore Singapore 119074 Singapore; ^4^ Department of Chemical and Biomolecular Engineering College of Design and Engineering National University of Singapore Singapore 117575 Singapore; ^5^ Department of Biomedical Engineering College of Design and Engineering National University of Singapore Singapore 117575 Singapore; ^6^ Department of Pharmacy and Pharmaceutical Sciences Faculty of Science National University of Singapore Singapore 117544 Singapore; ^7^ Clinical Imaging Research Centre Centre for Translational Medicine Yong Loo Lin School of Medicine National University of Singapore Singapore 117599 Singapore; ^8^ Nanomedicine Translational Research Program Yong Loo Lin School of Medicine National University of Singapore Singapore 117597 Singapore; ^9^ Theranostics Center of Excellence (TCE) Yong Loo Lin School of Medicine National University of Singapore Singapore 138667 Singapore; ^10^ Institute of Molecular and Cell Biology Agency for Science Technology and Research (A*STAR) Singapore 138673 Singapore

**Keywords:** drug delivery, lymphatic metastases, macrophage polarization, tumor immunotherapy

## Abstract

Lymphatic metastasis is a major cause of tumor treatment failure, with the immunosuppressive status of lymphatic macrophages significantly impairing antitumor immunity. In this study, it is found that CD169^+^ macrophages in lymphatic metastasis exhibit impaired phagocytic activity and diminished antigen‐presenting capacity, which correlates with suppressed antitumor immune responses. Based on these discoveries, a CD169^+^ macrophage‐targeted immunomodulator (designated as G‐LNP@S‐D) is fabricated to restore phagocytic function and enhance antigen presentation for lymphatic metastasis eradication. G‐LNP@S‐D consists of GM1‐functionalized liposomes co‐encapsulating the SHP2 inhibitor SHP099 and the STING agonist DMXAA, enabling sequential lymph node‐ and CD169^+^ macrophage‐specific drug delivery. Mechanistically, G‐LNP@S‐D not only restores the phagocytic capacity of CD169^+^ macrophages to eliminate tumor cells but also activates the STING pathway to enhance antigen presentation and subsequent T cell priming. Immunological profiling confirms that G‐LNP@S‐D treatment promotes the infiltration of CD4^+^ and CD8^+^ T cells in both TDLNs and primary tumors. Importantly, G‐LNP@S‐D exerts systemic immunomodulatory effects for directly eradicating lymphatic metastases. This study elucidates a sophisticated lymph node immune‐modulation strategy and provides a promising therapeutic approach to treat lymphatic metastasis.

## Graphical abstract

A CD169^+^ macrophage‐targeted immunomodulator (G‐LNP@S‐D) is developed to co‐deliver a SHP2 inhibitor and STING agonist, thereby restoring phagocytic function, enhancing antigen presentation by CD169^+^ macrophages, and promoting T cell priming to eradicate lymphatic metastasis.

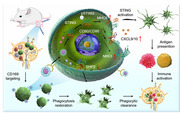

## 1 Introduction

Lymphatic metastasis remains one of the most formidable challenges in oncology, serving as both a prognostic indicator and therapeutic bottleneck for solid tumors.^[^ ^1^, ^2^, ^3^ ^]^ Mechanistically, the metastatic cascade begins when tumor cells infiltrate lymphatic vessels, subsequently colonizing sentinel lymph nodes (LNs) before disseminating hematogenously to distant organs.^[^ ^4^, ^5^, ^6^ ^]^ Clinically, this process correlates with a striking 30%‐50% reduction in 5‐year survival rates for malignancies including breast, prostate, and head‐neck cancers, while also predicting poor response to conventional therapies.^[^ ^7^ ^]^ Despite recent advances in targeted treatments and immunotherapy, metastatic LNs frequently persist as immunologically privileged sites that exhibit both intrinsic chemoresistance and remarkable immune evasion capabilities.^[^ ^8^, ^9^, ^10^, ^11^, ^12^, ^13^ ^]^ These clinical realities indicate the urgent need for effective strategies that target the unique biological features of lymphatic metastasis.

As central hubs of immune surveillance, LNs function as sophisticated immunological processing centers where intricate stromal networks coordinate optimal antigen presentation.^[^ ^14^, ^15^, ^16^, ^17^, ^18^, ^19^ ^]^ Within this network, lymphatic macrophages demonstrate remarkable functional specialization according to their anatomical positioning.^[^ ^20^, ^21^, ^22^, ^23^ ^]^ Particularly noteworthy are the subcapsular sinus macrophages (SSMs) and medullary sinus macrophages (MSMs), which are strategically positioned at the lymph‐tissue interface.^[^ ^24^, ^25^ ^]^ These specialized cells express pattern recognition receptors (CD169, MARCO, CD206) that facilitate efficient antigen capture.^[^ ^26^, ^27^, ^28^, ^29^, ^30^, ^31^ ^]^ Most importantly, CD169^+^ macrophages uniquely cross‐present tumor antigens to CD8^+^ T cells, thereby bridging innate and adaptive antitumor immunity.^[^ ^32^, ^33^, ^34^, ^35^, ^36^, ^37^ ^]^ However, metastatic transformation dramatically alters this protective mechanism, converting macrophages into immunosuppressive entities with both impaired phagocytic activity and defective antigen presentation capacity.^[^ ^14^, ^15^, ^38^, ^39^ ^]^ This functional impairment creates a permissive metastatic niche while simultaneously suppressing antitumor immune responses.^[^ ^16^ ^]^ Consequently, therapeutic targeting of lymphatic macrophages has emerged as a promising immunomodulatory strategy that capitalizes on their unique phagocytic functions and specialized antigen‐processing capabilities. Such a combinatorial approach could potentially transform metastatic LNs from immune‐privileged sanctuaries into active sites of antitumor immunity.

At the molecular level, tumor cells exploit the CD47‐signal regulatory protein α (SIRPα) axis to evade immune surveillance, wherein CD47 expression binds macrophage surface SIRPα to initiate an immunosuppressive cascade that potently inhibits phagocytic activity.^[^ ^40^, ^41^, ^42^ ^]^ This interaction specifically recruits and activates src homology 2 domain‐containing tyrosine phosphatase (SHP2) via its src homology 2 (SH2) domain, thereby amplifying the don“don't eat me” signal that enables tumor cells to escape macrophage‐mediated clearance.^[^ ^43^, ^44^, ^45^, ^46^, ^47^ ^]^ Complementing this pathway, the stimulator of interferon genes (STING) serves as a critical immunological bridge by activating TANK‐binding kinase 1 (TBK1)‐interferon regulatory factor 3 (IRF3) signaling through cytosolic DNA sensing (cGAS‐cGAMP).^[^ ^48^, ^49^, ^50^, ^51^, ^52^, ^53^ ^]^ This activation enhances antigen presentation through upregulation of both major histocompatibility complex (MHC) class I/II molecules and costimulatory markers (CD80/86).^[^ ^54^, ^55^, ^56^ ^]^ Therapeutically, simultaneous modulation of these pathways, combining SHP2 inhibition to restore phagocytic capacity with STING activation to enhance antigen presentation, represents a particularly promising immunomodulatory strategy for lymphatic macrophages. To maximize this synergistic effect, optimized drug formulations that enable both passive lymphatic drainage and active targeting of lymphatic macrophages are essential. Such an integrated approach, combining pathway‐specific modulators with advanced delivery platforms, may effectively overcome current therapeutic limitations in lymphatic metastasis by coordinately restoring critical immune surveillance functions.

**Scheme 1:**
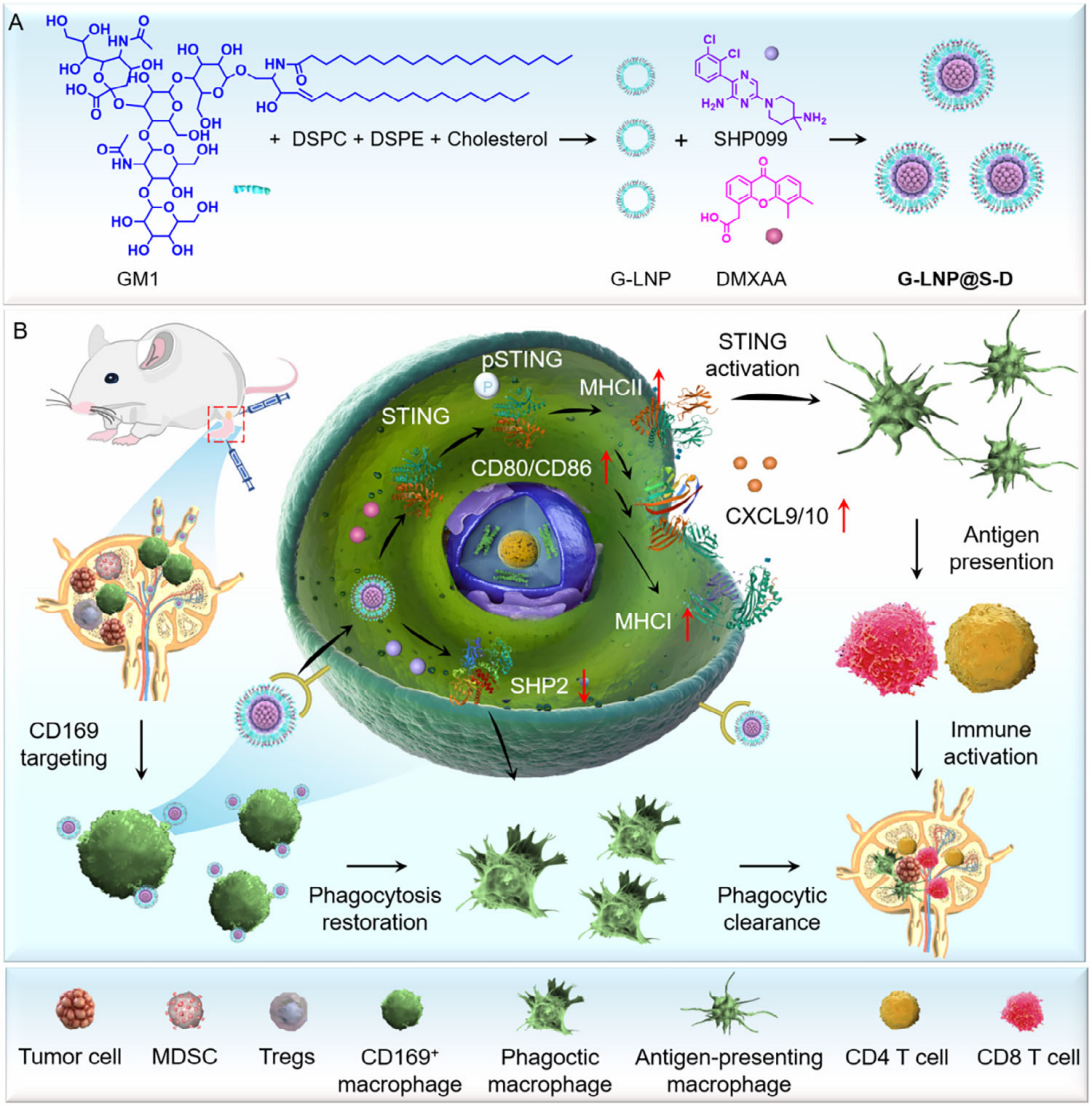
Fabrication and the proposed mechanism of G‐LNP@S‐D to restore phagocytic function and enhance antigen presentation for lymphatic metastasis eradication. A) Schematic illustration of the ganglioside 1 (GM1)‐functionalized liposomal formulation (G‐LNP@S‐D) co‐encapsulating the SHP2 inhibitor (SHP099) and STING agonist (DMXAA). B) Proposed immune activation mechanism of G‐LNP@S‐D for eradicating lymphatic metastasis. Upon subcutaneous administration, G‐LNP@S‐D sequentially targets CD169^+^ macrophages within metastatic lymph nodes (LNs). SHP099‐mediated SHP2 inhibition restores the phagocytic capacity of CD169^+^ macrophages, while DMXAA activates the STING pathway, enhancing antigen presentation. This dual modulation reactivates CD169^+^ macrophage function and promotes cytotoxic T cell activation, driving a synergistic immune response that effectively eliminates lymphatic metastases.

In this study, bioinformatic analysis combined with experimental validation revealed that CD169^+^ macrophages in lymphatic metastases exhibited impaired phagocytic activity and reduced antigen‐presenting capacity, contributing to suppressed antitumor immune responses. Based on these discoveries, a CD169^+^ macrophage‐targeted immunomodulator (designated as G‐LNP@S‐D) was fabricated to restore phagocytic function and enhance antigen presentation for the eradication of lymphatic metastases. G‐LNP@S‐D consisted of ganglioside 1 (GM1)‐functionalized liposomes co‐encapsulating the SHP2 inhibitor SHP099 and the STING agonist DMXAA (**Scheme** **1**A). Leveraging both the passive lymphatic drainage characteristic of nanomedicines and the sialic acid‐binding affinity of GM1, G‐LNP@S‐D efficiently accumulated in tumor‐draining lymph nodes (TDLNs) and is selectively internalized by CD169^+^ macrophages (Scheme 1B). Mechanistically, G‐LNP@S‐D reactivated the phagocytic function of CD169^+^ macrophages to facilitate tumor cell clearance, while simultaneously upregulating MHCI and MHCII expression to enhance antigen presentation and subsequent T cell priming. Immunological profiling demonstrated that G‐LNP@S‐D treatment promoted CD4^+^ and CD8^+^ T cell infiltration in both TDLNs and primary tumors, while reducing immunosuppressive populations of regulatory T cells (Tregs) and myeloid‐derived suppressor cells (MDSCs). Notably, G‐LNP@S‐D induced potent systemic immunomodulation, achieving direct elimination of lymphatic metastases. This work established a promising LN‐specific macrophage‐targeted immunomodulatory strategy, offering a novel therapeutic approach for the treatment of lymphatic metastasis.

## 2 Results and Discussion

### 2.1 Bioinformatics Analysis and Experimental Validation of CD169^+^ Macrophage in Breast Cancer

**Figure 1:**
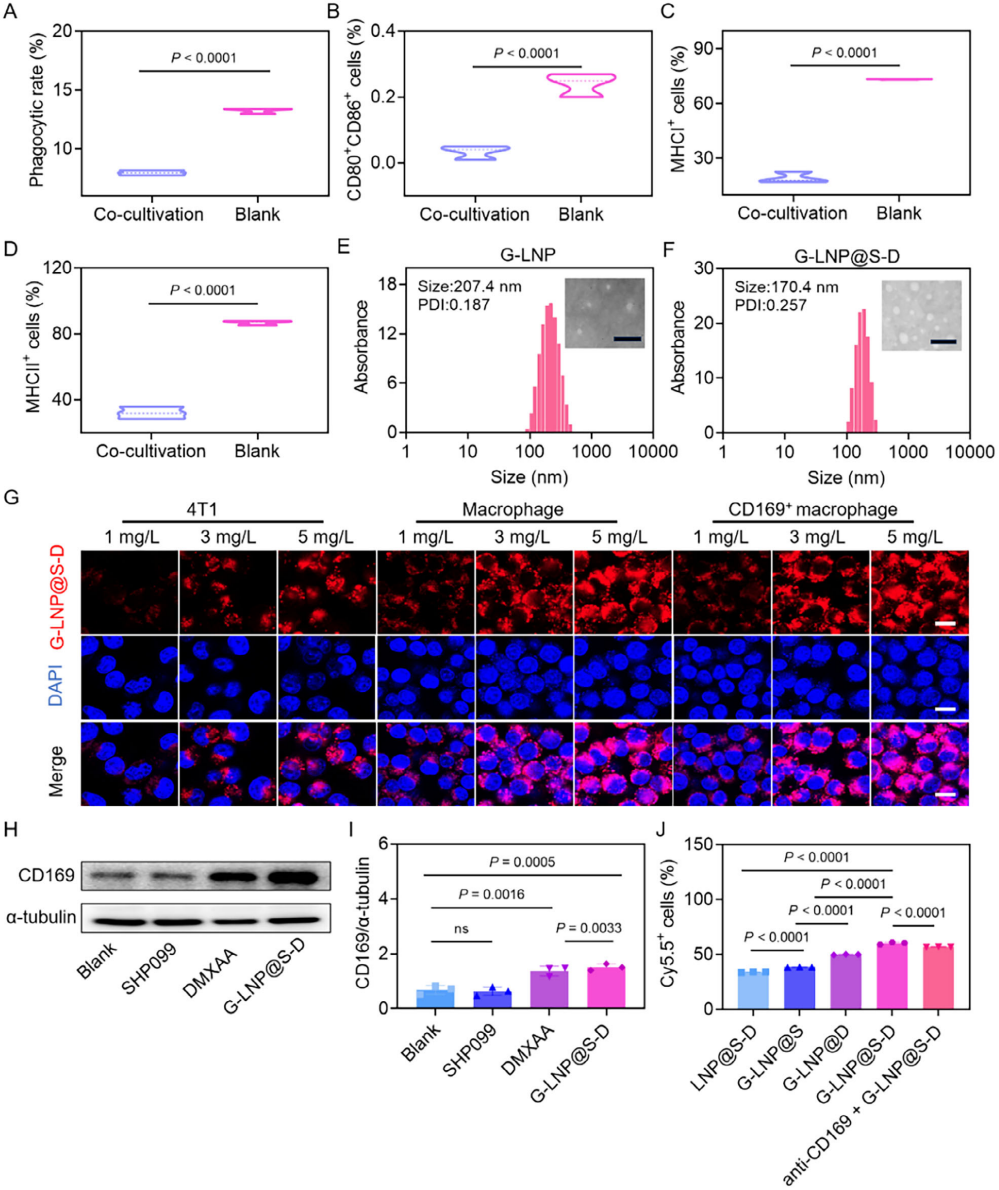
Mechanism verification of CD169^+^ macrophages and preparation of G‐LNP@S‐D for targeted drug co‐delivery. Flow cytometry quantification of the percentage of A) phagocytic cells, B) CD80^+^CD86^+^, C) MHCI^+^, and (D) MHCII^+^ macrophages in the co‐culture group (4T1 cells + CD169^+^ macrophages) versus the Blank group (CD169^+^ macrophages alone) (*n* = 3). Size distribution and TEM image of E) G‐LNP and F) G‐LNP@S‐D (scale bar: 500 nm). G) Confocal laser scanning microscope (CLSM) images of 4T1 cells, RAW264.7 macrophages, and CD169^+^ macrophages incubated with Cy5.5‐labeled G‐LNP@S‐D at varying concentrations (1, 3, 5 mg L^−1^). Scale bar: 10 µm. H) Western blot analysis of CD169 expression in CD169^+^ macrophages treated with SHP099, DMXAA, or G‐LNP@S‐D, and I) quantitative densitometry analysis. J) Flow cytometry analysis of cellular uptake behavior in CD169^+^ macrophages treated with LNP@S‐D, G‐LNP@S, G‐LNP@D, anti‐CD169 + G‐LNP@S‐D, or G‐LNP@S‐D. Statistical significance was determined using Student's t‐test or one‐way ANOVA.

Previous studies have identified CD169^+^ macrophages in LNs as being closely associated with poor prognosis in bladder cancer.^[^ ^57^ ^]^ Given the aggressive lymphatic metastatic behavior of breast cancer, we sought to investigate the relationship between CD169^+^ macrophages and breast cancer prognosis. CD169⁺ macrophages were differentiated from the bone marrow isolated from a BALB/c mouse (Figure S1A, Supporting Information). IFN‐α induction led to an upregulation of CD169 expression. Phenotypic characterization further identified distinct CD169⁺CD80⁺, and CD169⁺CD206⁺ subsets, implying a potential link to M1/M2 macrophage polarization that merited further investigation (Figure S1B, Supporting Information).^[^ ^21^ ^]^ Breast cancer cells strongly suppressed the phagocytic capacity of CD169⁺ macrophages, reducing the phagocytosis rate by 60% compared to the Blank group (**Figure** **1**A; Figure S1C, Supporting Information). Additionally, they significantly downregulated CD80⁺, CD86⁺, MHCI⁺, and MHCII⁺ expression on CD169⁺ macrophages by 7.2‐, 3.8‐, and 2.7‐fold, respectively (Figure 1B–D; Figure S1D–F, Supporting Information). These findings indicated that phenotypic changes in CD169^+^ macrophages might influence the immune status of breast cancer within LNs, and this factor might have been previously overlooked in breast cancer immunity. CD169 expression also positively correlated with tumor purity (Figure S1G, Supporting Information). Further analysis revealed positive associations between CD169 levels and infiltration of macrophages, CD8⁺ T cells, and CD4⁺ T cells, indicating a direct role in antitumor immunity (Figure S1H–J, Supporting Information). Together, these results demonstrated that CD169⁺ macrophages in LN metastases displayed diminished phagocytosis and impaired antigen presentation, fostering an immunosuppressive metastatic niche.

### 2.2 Preparation and Characterization of G‐LNP@S‐D

Given the impaired phagocytosis and antigen presentation of CD169⁺ macrophages in breast cancer, we designed a targeted combination strategy to restore these functions. We utilized the SHP2 inhibitor SHP099, which enhanced macrophage phagocytosis by disrupting the CD47‐SIRPα axis, and the murine STING agonist DMXAA, known to promote inflammatory cytokine release and antigen presentation.^[^ ^43^, ^45^, ^48^, ^49^, ^58^ ^]^ To enable LN‐specific delivery, the CD169‐targeting ligand GM1 was incorporated into liposomal carriers for precise targeting of CD169⁺ macrophages.^[^ ^59^, ^60^ ^]^ Therefore, GM1‐modified liposomes (G‐LNP) and SHP099/DMXAA‐encapsulated G‐LNP (G‐LNP@S‐D) were systematically optimized by screening various feed ratios of lipid components and drug loading ratios. A DSPC:cholesterol:DSPE:GM1 ratio of 11:7:1:1 yielded uniform and stable nanoparticles (Figure 1E; Figure S2A–G, Supporting Information). Further adjustment of lipid:SHP099:DMXAA ratios produced G‐LNP@S‐D with uniform size and efficient drug encapsulation (Figure 1F; Figure S2H–J, Supporting Information). A feed ratio of 170:5:18 conferred stability in aqueous solution for 7 days (Figure S2K–N, Supporting Information). Both G‐LNP and G‐LNP@S‐D exhibited negative zeta potentials, supporting colloidal stability (Figure S3, Supporting Information). High‐performance liquid chromatography (HPLC) analysis revealed that the loading efficiencies of SHP099 and DMXAA in G‐LNP@S‐D were 74.0 ± 3.0% and 64.2 ± 3.2%, respectively (Figure S4A–C, Supporting Information). Drug release was accelerated at pH 5.5 compared to pH 7.4, favoring rapid drug activity in the tumor microenvironment (Figure S5, Supporting Information). These results demonstrated the successful preparation of G‐LNP@S‐D, supporting its potential for targeted therapy of lymphatic metastases.

### 2.3 Macrophage Targeting Behavior of G‐LNP@S‐D

First, the targeting specificity of G‐LNP@S‐D was evaluated in 4T1 tumor cells, RAW264.7 macrophages, and CD169⁺ macrophages. G‐LNP@S‐D showed concentration‐dependent cellular uptake in all cell types, with the highest level in CD169⁺ macrophages (Figure 1G; Figure S6, Supporting Information). Western blot analysis revealed that DMXAA and G‐LNP@S‐D upregulated CD169 expression by 2.1‐ and 2.3‐fold, respectively, compared to the Blank group, suggesting that DMXAA promoted uptake via CD169 induction (Figure 1H–I). When comparing different formulations, G‐LNP@S‐D exhibited the strongest fluorescence signal in CD169⁺ macrophages, while non‐targeted LNP@S‐D showed the lowest uptake (Figure 1J). Pre‐incubation with an anti‐CD169 antibody reduced cellular uptake by 8%, further confirming CD169‐mediated targeting. Cy5.5‐labeled G‐LNP@S‐D was co‐incubated with anti‐CD169‐stained CD169⁺ macrophages to assess their co‐localization. As shown in Figure S7A (Supporting Information), the red fluorescence of G‐LNP@S‐D overlapped with the green CD169 signal, producing a distinct yellow signal. Flow cytometry further confirmed concentration‐dependent phagocytosis, with the phagocytic rate at 5 mg L^−1^ being 1.51‐fold and 13.6‐fold higher than at 3 and 1 mg L^−1^, respectively (Figure S7B,C, Supporting Information). These results demonstrated that G‐LNP@S‐D effectively targeted CD169⁺ macrophages via GM1, while drug‐induced CD169 upregulation established a positive feedback loop that enhanced cellular uptake and delivery efficiency.

### 2.4 Phagocytic Restoration and Tumor Cell Elimination Abilities of G‐LNP@S‐D

**Figure 2:**
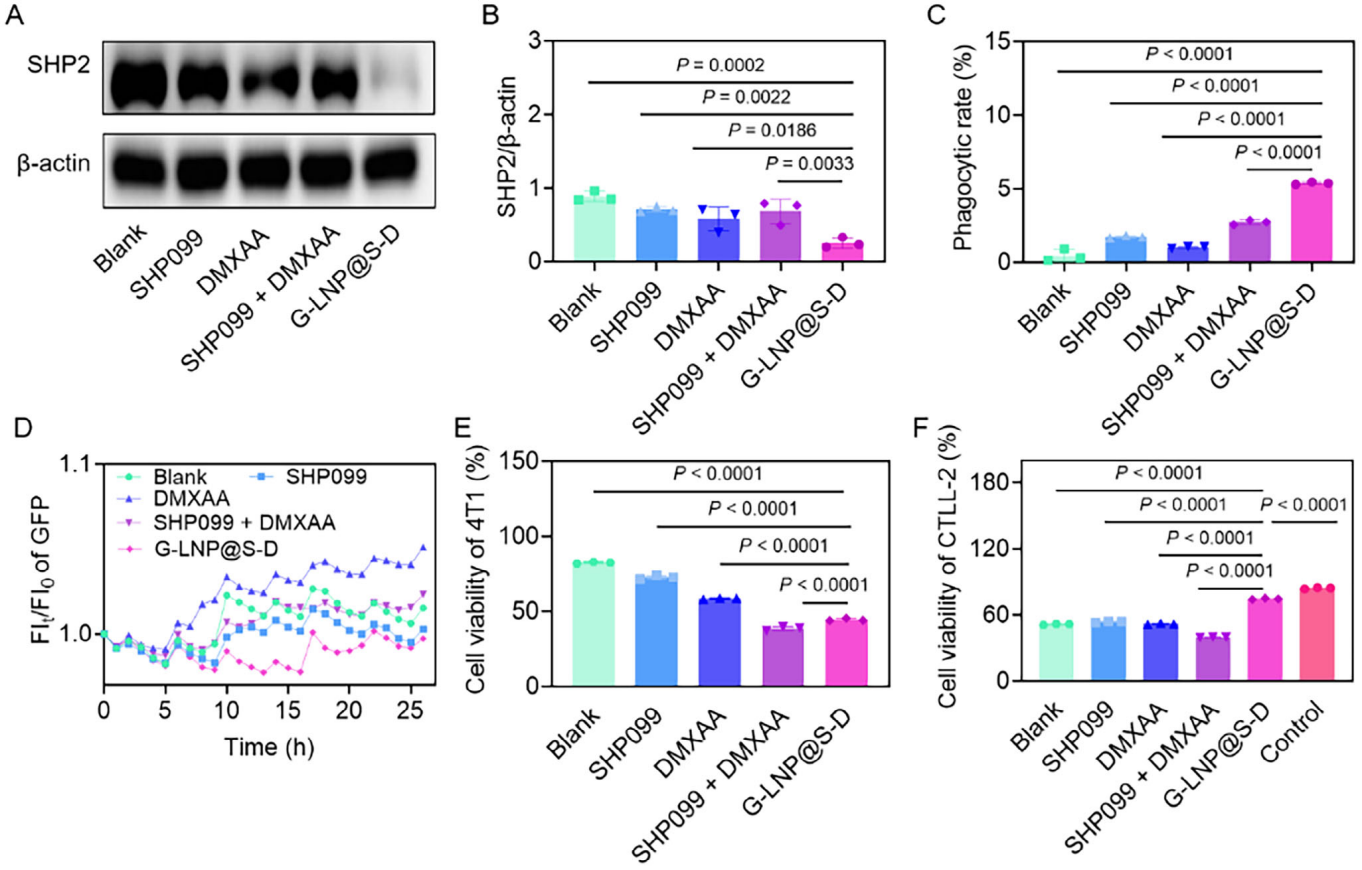
Restoration of phagocytic activity and tumor cell elimination by CD169^+^ macrophages following G‐LNP@S‐D treatment. A) Western blot analysis and B) quantitative densitometry of SHP2 expression levels in CD169^+^ macrophages following treatment with SHP099, DMXAA, SHP099 + DMXAA, or G‐LNP@S‐D. C) Flow cytometry quantification of the phagocytic clearance of 4T1 tumor cells by CD169^+^ macrophages after indicated treatments. D) Time‐dependent fluorescence signal from 4T1‐GFP cells co‐cultured with CD169^+^ macrophages treated with SHP099, DMXAA, SHP099 + DMXAA, or G‐LNP@S‐D over 26 h, indicating dynamic phagocytic elimination. E) Corresponding viability of apoptosis analysis of 4T1 cells via PI/Annexin V‐FITC staining. F) Viability quantification of CTLL‐2 cytotoxic T cells to assess cytotoxicity of drug regimens on immune effector cells. Statistical comparisons were performed using one‐way ANOVA.

The cytotoxic effects of G‐LNP@S‐D were assessed in 4T1 tumor cells and CD169⁺ macrophages. Cell viability remained above 90% across all treatment groups, indicating no direct cytotoxicity to either cell type (Figure S8A,B, Supporting Information). Notably, G‐LNP@S‐D downregulated proteins in the SHP2‐mediated phagocytosis pathway (**Figure** **2**A,B). SHP2 expression was reduced to 80.0%, 79.5%, and 19.8% of the Blank group by SHP099, SHP099 + DMXAA, and G‐LNP@S‐D, respectively. Functional assays confirmed that G‐LNP@S‐D strongly enhanced phagocytosis of GFP‐labeled 4T1 cells by CD169⁺ macrophages (Figure S8C, Supporting Information). Quantification revealed phagocytic rates were 2.0‐, 1.2‐, 3.2‐, and 6.0‐fold higher with SHP099, DMXAA, SHP099 + DMXAA, and G‐LNP@S‐D, respectively, compared to the Blank group (Figure 2C). Confocal laser scanning microscope (CLSM) revealed enhanced tumor cell engulfment by CD169⁺ macrophages following G‐LNP@S‐D treatment. While only one tumor cell was internalized in the Blank group, multiple GFP⁺ tumor cells were phagocytosed after treatment, with 3D imaging confirming co‐localization (Figure S8D, Supporting Information). This phenomenon indicated that G‐LNP@S‐D promoted phagocytosis by inhibiting SHP2 and blocking the CD47‐SIRPα pathway. Real‐time imaging further showed internalization and progressive degradation of 4T1‐GFP fluorescence in CD169⁺ macrophages. Over 25 h, fluorescence exhibited a wave‐like decline across groups, with the most pronounced reduction in the G‐LNP@S‐D group, reaching a minimum at 16 h (Figure 2D). Despite partial signal recovery, this group maintained the lowest overall fluorescence, confirming superior tumoricidal efficacy. Collectively, these results demonstrated that G‐LNP@S‐D effectively restored the phagocytic function of CD169⁺ macrophages and enhanced tumor cell clearance.

Beyond phagocytosis, macrophages also act as antigen‐presenting cells that activate cytotoxic T cells to exert tumoricidal effects.^[^ ^61^ ^]^ To evaluate the immunomodulatory function of G‑LNP@S‑D, a triple co‑culture system of 4T1 cells, CD169⁺ macrophages, and CTLL‑2 T cells was established. G‑LNP@S‑D significantly reduced tumor cell viability in this system (Figure S8E, Supporting Information). Specifically, the survival rates of 4T1 cells in the Blank, SHP099, DMXAA, SHP099 + DMXAA, and G‐LNP@S‐D groups were 82.3%, 73.4%, 58.7%, 38.7%, and 44.3%, respectively, indicating that G‑LNP@S‑D suppresses tumor proliferation via coordinated macrophage and T‑cell activity (Figure 2E). Nevertheless, tumor cells can impair T‑cell function through diverse immunosuppressive mechanisms.^[^ ^62^, ^63^ ^]^ Notably, G‐LNP@S‐D maintained CTLL‐2 T‐cell viability at levels comparable to T‐cells alone (Figure S8F, Supporting Information). The viability of CTLL‐2 cells in the Blank, SHP099, DMXAA, SHP099 + DMXAA, and G‐LNP@S‐D groups was 51.7%, 45.6%, 53.1%, 39.7%, and 74.0%, respectively (Figure 2F). Furthermore, co‐culture with tumor cells upregulated the T‐cell exhaustion marker TIM‐3 by 47.2% compared to T‐cells alone (Figure S9A,B, Supporting Information). Treatment with SHP099, DMXAA, SHP099 + DMXAA, and G‐LNP@S‐D reduced TIM‐3 expression by 94.9%, 92.8%, 89.6%, and 88.1%, respectively. These results indicated that G‐LNP@S‐D enhanced antitumor immunity while preserving T‐cell viability and mitigating T‐cell exhaustion in the tumor microenvironment.

### 2.5 STING Activation and Antigen Presentation Enhancement by G‐LNP@S‐D

**Figure 3:**
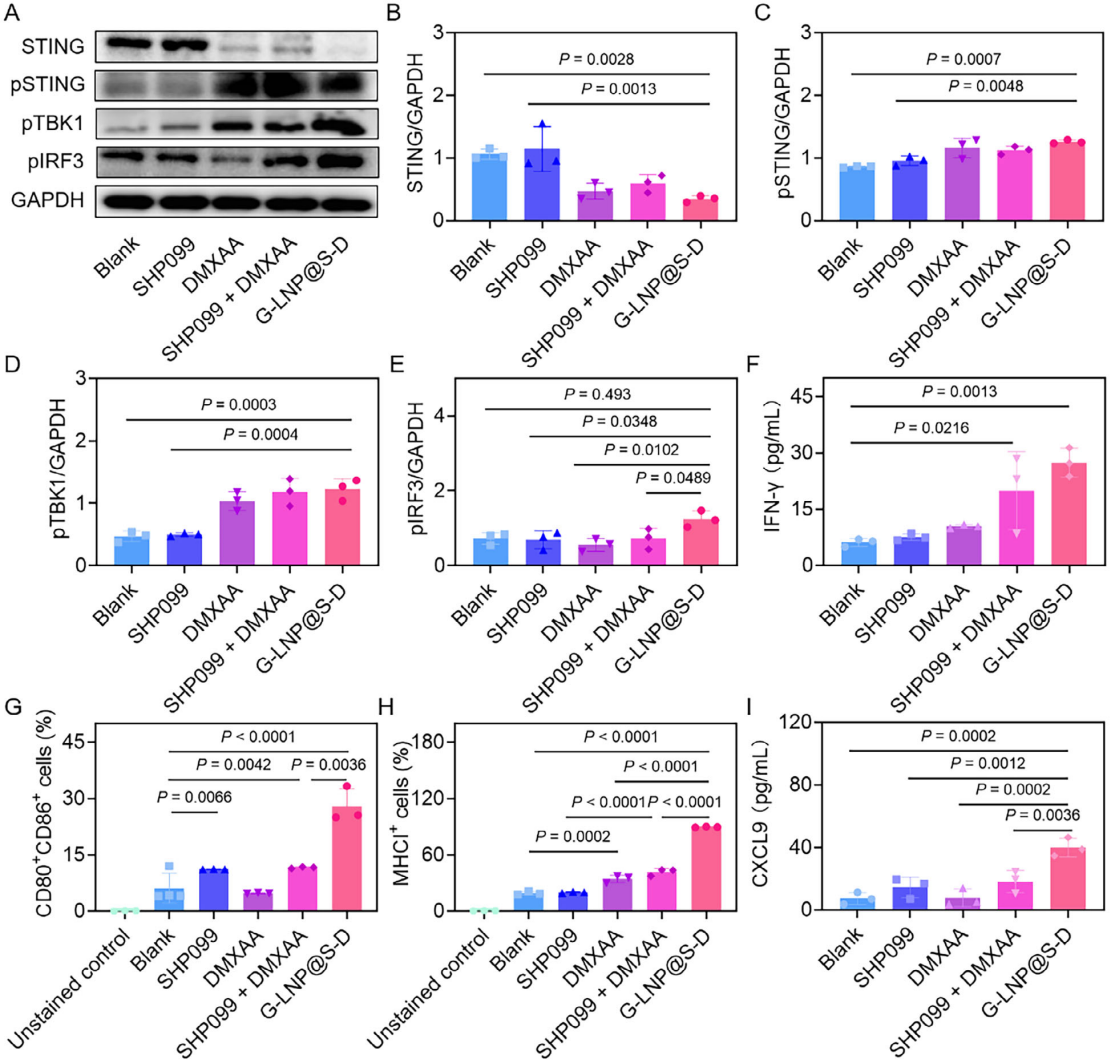
Activation of STING signaling and enhancement of antigen presentation by G‐LNP@S‐D in CD169^+^ macrophages. A) Western blot analysis of the expression of STING, pSTING, pTBK1, and pIRF3 in CD169^+^ macrophages following treatment with SHP099, DMXAA, SHP099 + DMXAA, or G‐LNP@S‐D. Quantitative densitometry of B) STING, C) pSTING, D) pTBK1, and E) pIRF3 in CD169^+^ macrophages based on western blot analysis. F) The expression of IFN‐γ after treatment with SHP099, DMXAA, SHP099 + DMXAA or G‐LNP@S‐D. Flow cytometry quantification of antigen presentation markers on CD169^+^ macrophages including G) CD80^+^CD86^+^ or H) MHCI^+^ after treatment with SHP099, DMXAA, SHP099 + DMXAA or G‐LNP@S‐D. I) The expression of C‐X‐C motif chemokine ligand 9 (CXCL9) on CD169^+^ macrophages after treatment with SHP099, DMXAA, SHP099 + DMXAA or G‐LNP@S‐D. P values were tested via a one‐way ANOVA analysis.

Subsequently, the immunomodulatory effect of G‐LNP@S‐D on STING pathway activation in macrophages was evaluated. DMXAA‐containing formulations markedly altered the expression of STING pathway proteins, including STING, pSTING, pTBK1, and pIRF3 (**Figure** **3**A). Total STING expression decreased to 43.7%, 53.1%, and 40.6% in the DMXAA, SHP099 + DMXAA, and G‐LNP@S‐D groups, respectively, consistent with activation‐induced degradation (Figure 3B). In contrast, G‐LNP@S‐D treatment induced the highest upregulation of pSTING, pTBK1, and pIRF3‐1.4‐, 2.6‐, and 1.8‐fold over the Blank group, confirming robust STING pathway activation (Figure 3C–E).

Correspondingly, STING activation significantly enhanced type I interferon production, with IFN‐α, IFN‐β, and IFN‐γ markedly upregulated in macrophages. IFN‐γ showed the most pronounced increase (Figure 3F; Figure S10A,B, Supporting Information). G‐LNP@S‐D increased IFN‐γ levels by 4.5‐, 3.6‐, 2.6‐, and 1.4‐fold over Blank, SHP099, DMXAA, and SHP099 + DMXAA, respectively. G‐LNP@S‐D also enhanced antigen presentation in CD169⁺ macrophages. CD80⁺CD86⁺ expression increased 2.8‐, 1.2‐, and 3.0‐fold with SHP099, DMXAA, and SHP099 + DMXAA, respectively, and 7.0‐fold with G‐LNP@S‐D (Figure 3G; Figure S10C, Supporting Information). Similarly, MHC class I and II expression was also enhanced, with G‐LNP@S‐D increasing MHCI and MHCII levels by 4.7‐ and 1.6‐fold, respectively (Figure 3H; Figure S10D–F, Supporting Information). Additionally, chemokine secretion was assessed to determine the potential for T‐cell recruitment. G‐LNP@S‐D‐treated macrophages had substantial increases in C‐X‐C motif chemokine ligand 9 (CXCL9) and CXCL10 secretion‐5.5‐ and 2.2‐fold, respectively, indicating enhanced T‐cell chemoattraction potential (Figure 3I; Figure S10G, Supporting Information). In summary, G‐LNP@S‐D effectively activated the STING‐TBK1‐IRF3 signaling axis in macrophages, thereby promoting type I interferon production, upregulating MHC and co‐stimulatory molecule expression, and enhancing chemokine‐mediated T‐cell recruitment. These findings implied the potential of G‐LNP@S‐D to potentiate antigen presentation and facilitate robust T‐cell‐mediated antitumor immunity.

### 2.6 LN Targeting and Inhibition of Lymphatic Metastasis by G‐LNP@S‐D In Vivo

**Figure 4:**
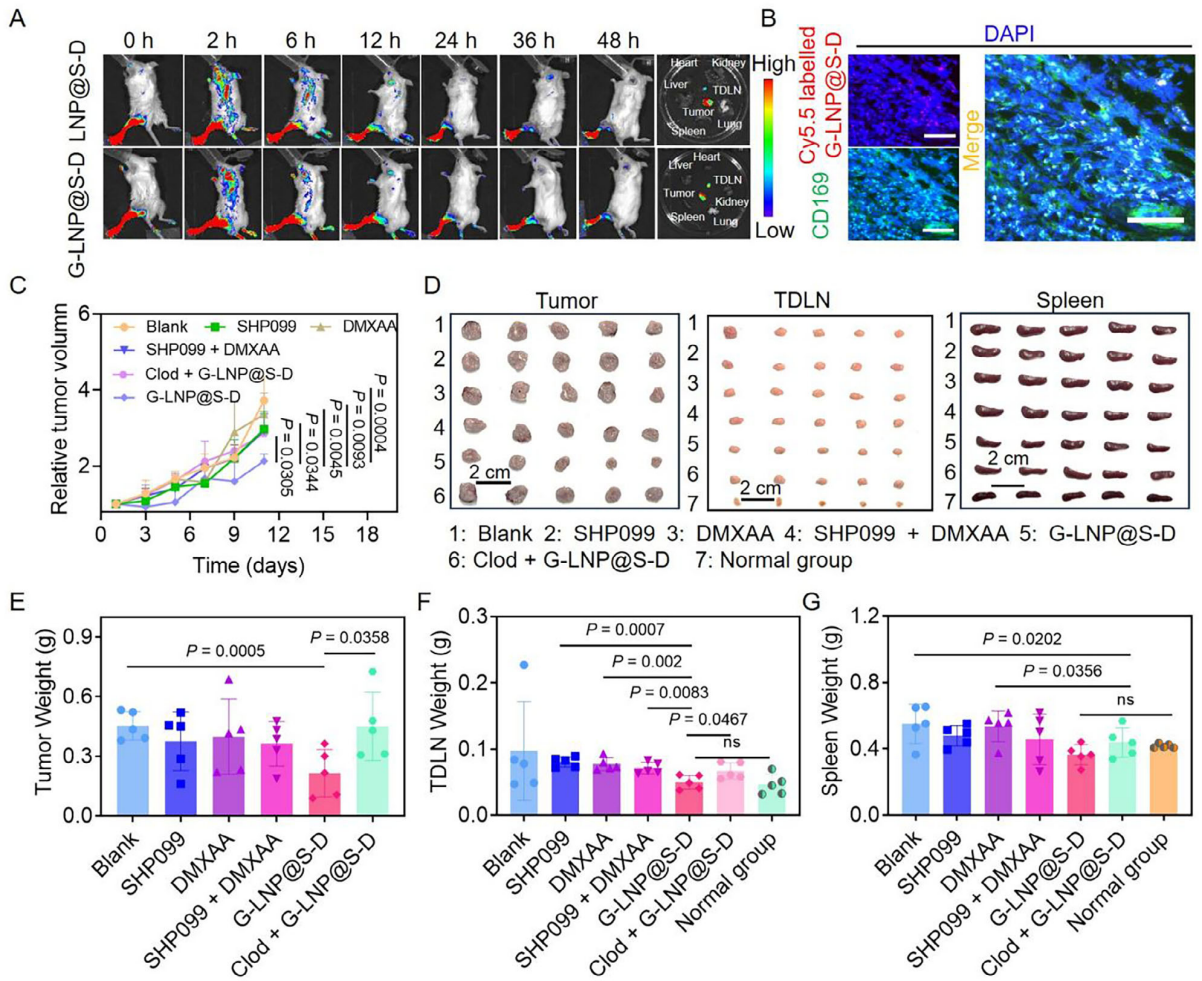
Lymph nodes (LNs) targeted G‐LNP@S‐D augments antitumor efficacy in lymphatic metastasis. A) In vivo fluorescence imaging of 4T1 tumor‐bearing mice following intravenous injection of LNP@S‐D or G‐LNP@S‐D at various time points (0, 2, 6, 12, 24, 36, and 48 h), and corresponding *ex vivo* organ imaging at 48 h post‐injection. B) CLSM images showing the specific uptake of G‐LNP@S‐D by LN‐resident macrophages 1 h post‐injection. Scale bar: 100 µm. C) Relative tumor volume progression in 4T1 tumor‐bearing mice subjected to various treatments. D) The representative images of tumor, TDLN, and spleen harvested on day 11 following administration of SHP099, DMXAA, SHP099 + DMXAA, G‐LNP@S‐D, or clodronate liposome (Clod) + G‐LNP@S‐D. Quantitative weight measurements of E) tumors, F) TDLNs, and G) spleens collected post‐treatment with SHP099, DMXAA, SHP099 + DMXAA, G‐LNP@S‐D, or Clod + G‐LNP@S‐D on the 11th day. Statistical significance was assessed using one‐way ANOVA.

A lymphatic metastasis model was established to evaluate the LN‐targeting capacity of G‑LNP@S‑D. By day 9, metastasis to popliteal LNs was confirmed upon excision and imaging of primary footpad tumors and corresponding LNs (Figure S11A, Supporting Information). After subcutaneous injection of Cy5.5‑labeled G‐LNP@S‐D or LNP@S‐D formulations, in vivo imaging revealed efficient accumulation at the primary tumor and in popliteal LNs for up to 48 h, with minimal off‑target organ distribution (**Figure** **4**A). Although both LNP@S‑D and G‑LNP@S‑D accumulated in tumors due to optimized particle size, G‑LNP@S‑D showed markedly enhanced LN retention, demonstrating GM1‑mediated targeting (Figure S11B, Supporting Information). Immunofluorescence of harvested LNs confirmed significant colocalization of G‑LNP@S‑D with CD169⁺ macrophages (Figure 4B). These results proved that G‑LNP@S‑D enabled sequential targeting of LNs and CD169⁺ macrophages, supporting localized immunomodulation against lymphatic metastasis.

Based on the potent in vitro anti‐proliferative activity and favorable lymphatic biodistribution of G‐LNP@S‐D, its therapeutic efficacy was evaluated in a footpad tumor‐bearing mouse model. Tumor volumes increased across all groups over time, yet G‐LNP@S‐D treatment significantly delayed tumor progression (Figure 4C; Figure S11C–H, Supporting Information). At the study endpoint, excised primary tumors, popliteal LNs, and spleens revealed that the G‐LNP@S‐D group exhibited the smallest tumor burden, along with reduced LN and spleen sizes (Figure 4D). Quantitatively, G‐LNP@S‐D reduced primary tumor mass by 54.6%, LN weight by 56.7%, and spleen weight by 34.5% compared to the Blank group (Figure 4E–G), confirming its efficacy in suppressing both primary and metastatic tumor progression.

To confirm macrophage involvement in the anti‐tumor response, clodronate liposomes (Clod) were used for macrophage depletion. This depletion markedly reduced the efficacy of G‐LNP@S‐D, underscoring the essential role of macrophages in its therapeutic effect. Hematoxylin and eosin (H&E) staining revealed substantial cellular atrophy and necrosis in treatment groups, most prominent with G‐LNP@S‐D; this effect was diminished by Clod pretreatment (Figure S11I, Supporting Information). TUNEL staining further showed G‐LNP@S‐D enhanced apoptosis in primary tumors and LN metastases by 1.2‐ and 2.5‐fold, respectively, over the Blank group (Figure S11J–M, Supporting Information). These findings demonstrated that G‐LNP@S‐D suppressed tumor growth and lymphatic metastasis by reprogramming CD169⁺ macrophages in LNs, thereby restoring anti‐tumor immunity and presenting a promising approach against metastatic tumors.

### 2.7 Immune Activation Effects of G‐LNP@S‐D In Vivo

**Figure 5:**
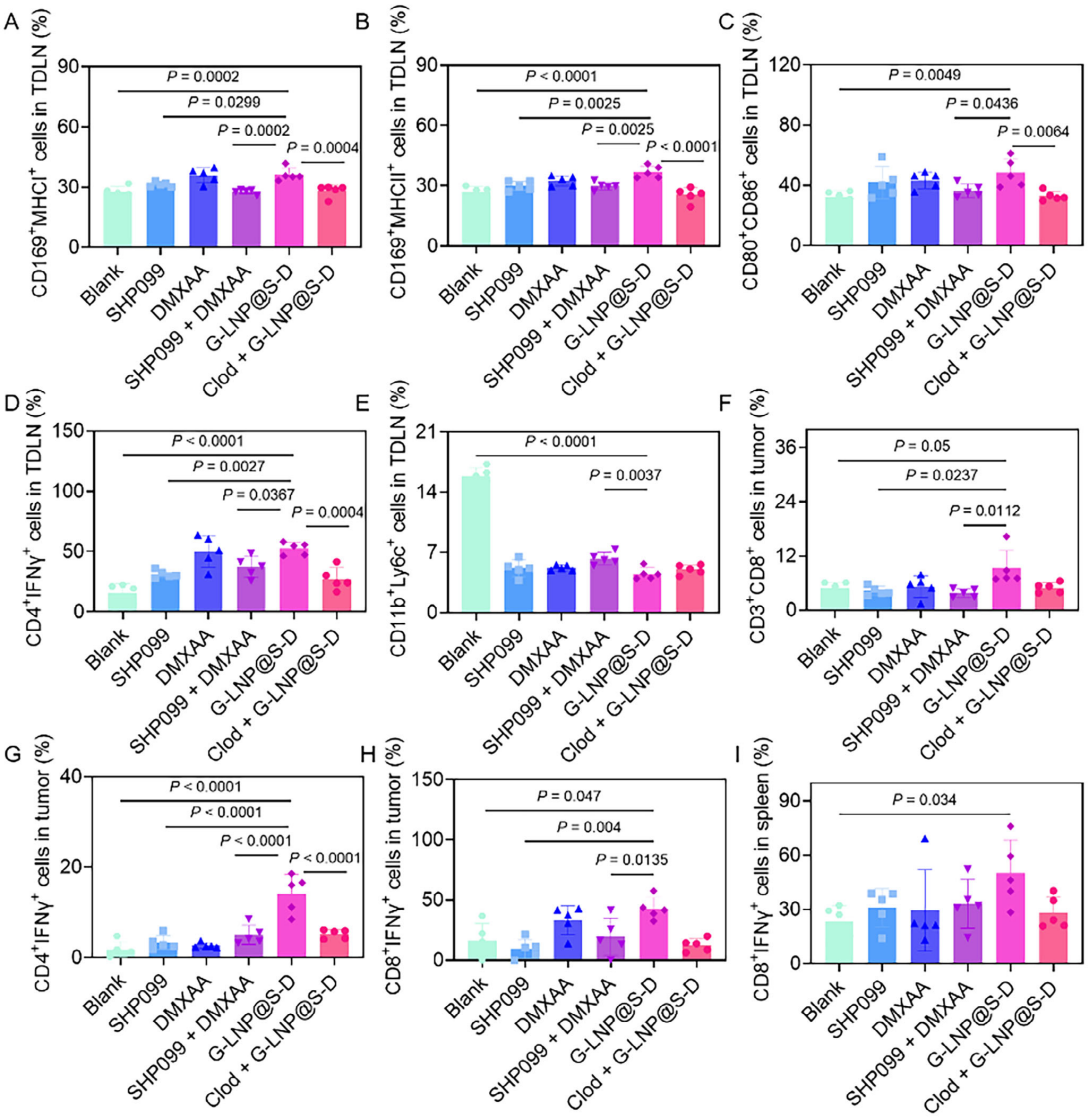
Immune activation and systemic remodeling induced by G‐LNP@S‐D. The percentages of A) CD169^+^MHCI^+^, B) CD169^+^MHCII^+^, C) CD80^+^CD86^+^ macrophages, D) CD4^+^IFNγ^+^ T lymphocytes, E) CD11b^+^Ly6c^+^ cells in TDLN after treatment with SHP099, DMXAA, SHP099 + DMXAA, G‐LNP@S‐D or Clod + G‐LNP@S‐D. The percentages of F) CD3^+^CD8^+^ T lymphocytes, G) CD4^+^IFNγ^+^ T lymphocytes, and (H) CD8^+^IFNγ^+^ T lymphocytes in tumors after treatment with SHP099, DMXAA, SHP099 + DMXAA, G‐LNP@S‐D or Clod + G‐LNP@S‐D. I) Percentages of CD8^+^IFNγ^+^ T lymphocytes in spleens after treatment with SHP099, DMXAA, SHP099 + DMXAA, G‐LNP@S‐D, or Clod + G‐LNP@S‐D. Statistical comparisons were performed using one‐way ANOVA.

The potent anti‐metastatic effect of G‐LNP@S‐D prompted investigation into its immunomodulation within TDLNs. Flow cytometry showed that SHP099, DMXAA, SHP099+DMXAA, and G‐LNP@S‐D increased CD169⁺MHCI⁺ macrophage populations by 1.1‐, 1.3‐, 1.1‐, and 1.3‐fold, respectively, versus Blank (**Figure** **5**A; Figure S12, Supporting Information). Similarly, CD169⁺MHCII⁺ macrophages rose by 1.1‐, 1.2‐, 1.1‐, and 1.4‐fold (Figure 5B; Figure S13, Supporting Information). These increases were abolished in the Clod + G‐LNP@S‐D group, confirming macrophage‐dependent effects. Concurrently, co‐stimulatory molecules (CD80^+^CD86^+^) expression was elevated by 30.4%, 34.8%, 13.7%, and 50.3% across treatment groups, indicating that G‐LNP@S‐D enhanced antigen presentation via MHC and co‐stimulatory molecule upregulation on CD169⁺ macrophages in vivo (Figure 5C; Figure S14, Supporting Information). Immunophenotyping of LN‐resident T cells revealed increased CD4⁺IFN‐γ⁺ and CD8⁺IFN‐γ⁺ populations across treatments, with G‐LNP@S‐D and DMXAA showing the greatest enhancement (Figure 5D; Figures S15 and S16A,B, Supporting Information). Mechanistically, DMXAA in G‐LNP@S‐D activated the STING pathway, inducing type I interferon production that reprogrammed MDSCs into mature macrophages or dendritic cells (DCs). This shift downregulated arginase‐1 (Arg‐1) and reactive oxygen species (ROS) via signal transducer and activator of transcription 1 (STAT1), reducing immunosuppression, while curbing MDSC recruitment through suppressed C‐C motif ligand 2 (CCL2) and CXCL12. Concurrently, interferon signaling downregulated forkhead box P3 (FoxP3) to inhibit Tregs differentiation and indirectly bolstered CD8⁺ T and NK cell cytotoxicity.^[^ ^64^ ^]^

**Figure 6:**
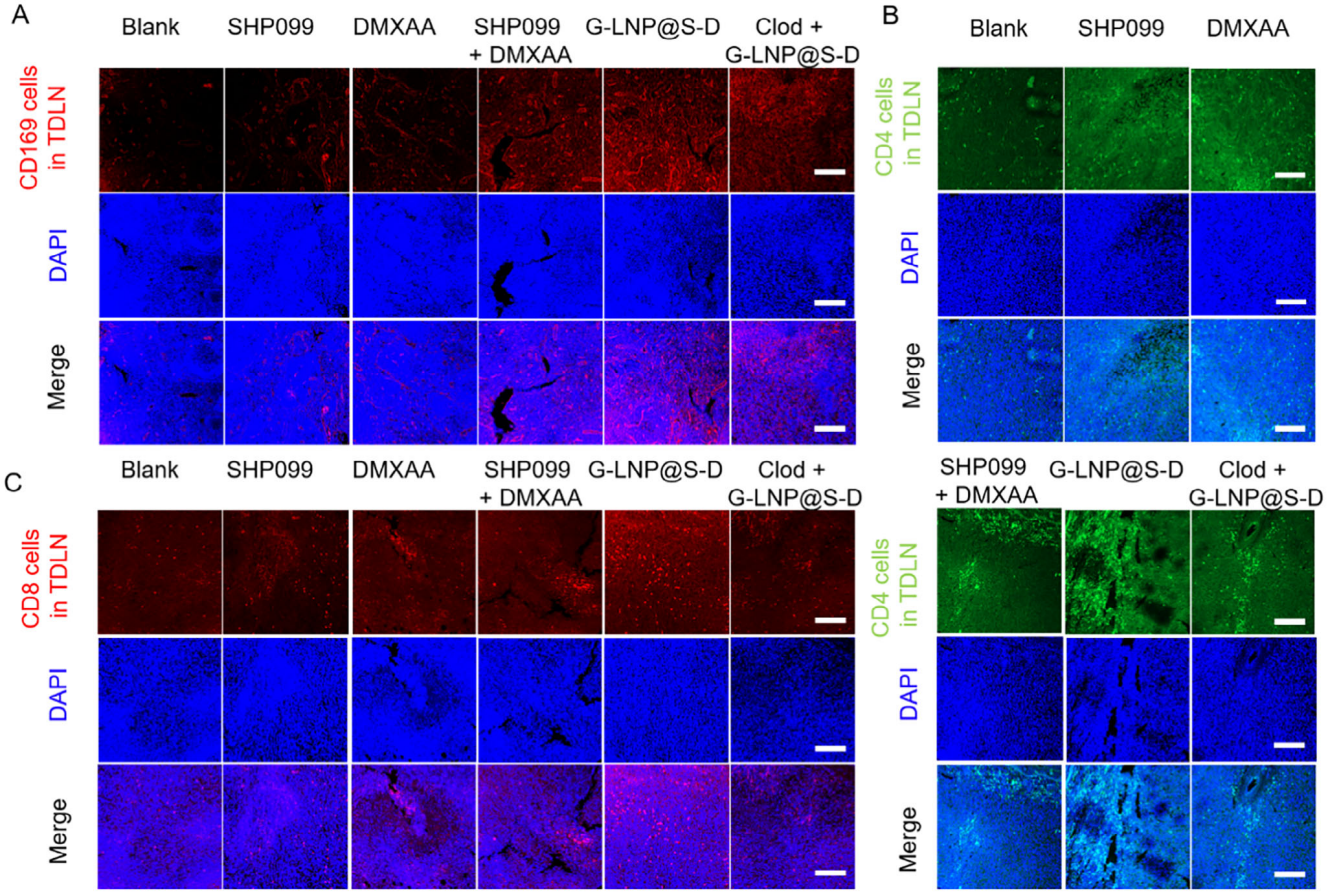
In vivo immunomodulatory effects and biosafety evaluation of G‐LNP@S‐D. Immunofluorescent staining of A) CD169^+^ macrophages, B) CD4^+^ T cells, and C) CD8^+^ T cells in TDLNs collected from 4T1 tumor‐bearing mice on day 11 following treatment with SHP099, DMXAA, SHP099 + DMXAA, G‐LNP@S‐D, or Clod + G‐LNP@S‐D. Scale bar: 100 µm.

Consequently, G‐LNP@S‐D substantially reduced immunosuppressive cells in TDLNs. CD11b⁺Ly6c⁺ MDSCs decreased by 32.2%, 32.7%, 39.2%, and 71.6% in the SHP099, DMXAA, SHP099 + DMXAA, and G‐LNP@S‐D groups, respectively, while Tregs (CD4⁺Foxp3⁺) fell to 48.8% of baseline with G‐LNP@S‐D (Figure 5E; Figures S17 and S18A,B, Supporting Information). In footpad tumors, G‐LNP@S‐D increased infiltration of CD3⁺CD4⁺ and CD3⁺CD8⁺ T cells by 5.9‐ and 1.9‐fold, respectively, and boosted IFN‐γ⁺ CD4⁺ and CD8⁺ T cells by 7.9‐ and 2.6‐fold (Figure 5F–H; Figures S19A,B and S20–S22, Supporting Information). Systemic immune activation was also observed, with splenic CD3⁺CD4⁺, and CD8⁺IFN‐γ⁺ T cells rising 1.27‐ and 2.1‐fold (Figure 5I; Figures S23A,B and S24, Supporting Information). Immunofluorescence of LNs confirmed strongest upregulation of CD169, CD4, and CD8 with G‐LNP@S‐D (**Figure** **6**A–C). G‐LNP@S‐D increased CD169⁺ macrophage infiltration in LNs by 3.5‐fold versus Blank, which declined to 56.6% upon macrophage depletion (Figure S25A, Supporting Information). Infiltration of CD4⁺ and CD8⁺ T cells also rose by 1.9‐ and 2.6‐fold, respectively (Figure S25B,C, Supporting Information). Collectively, G‐LNP@S‐D remodeled the metastatic lymphatic niche by enhancing antigen presentation and reversing immunosuppression via CD169⁺ macrophage reprogramming, thereby boosting local and systemic T cell‐mediated antitumor immunity.

### 2.8 Biosafety Evaluation of G‐LNP@S‐D

To evaluate the biosafety of the treatment regimens, histopathological and hematological analyses were performed. H&E staining of major organs, including the heart, liver, spleen, lungs, and kidneys, revealed no observable pathological abnormalities in mice treated with SHP099, DMXAA, SHP099 + DMXAA, or G‐LNP@S‐D, with tissue architectures remaining largely intact (Figure S26A, Supporting Information). In parallel, serum biochemical analyses were conducted to assess systemic toxicity. All measured parameters, including liver function markers (aspartate aminotransferase (AST) and alanine aminotransferase (ALT)) and renal function indicators (urea (UREA), uric acid (UA), and creatinine (CREA)), remained within physiological ranges across all treatment groups, suggesting preserved hepatic and renal function (Figure S26B, Supporting Information). Furthermore, body weights were monitored throughout the therapeutic course, with all groups exhibiting stable weights and fluctuations confined within an acceptable range (Figure S26C, Supporting Information). Collectively, these results indicated that G‐LNP@S‐D possessed excellent biocompatibility and induced no discernible systemic toxicity in vivo.

## 3 Conclusion

In summary, this study uncovered a significant association between the immunosuppressive phenotype of CD169^+^ macrophages and the progression of lymphatic metastatic tumors. Leveraging these insights, we rationally developed a CD169^+^ macrophage‐targeting immunomodulator (G‐LNP@S‐D) aimed at restoring phagocytic elimination and enhancing antigen presentation in CD169^+^ macrophages for the effective eradication of lymphatic metastatic tumors. Surface modification with GM1 conferred G‐LNP@S‐D with precise tropism for LNs and selective accumulation in CD169^+^ macrophages, thereby ensuring targeted delivery and reducing systemic exposure. Mechanistically, SHP2 inhibition reinstated macrophage‐mediated phagocytosis, while concurrent activation of the STING pathway amplified type I interferon signaling, promoting the antigen‐presenting function of CD169^+^ macrophages. This dual‐pronged immunomodulation synergistically reactivated both innate and adaptive antitumor immune responses, ultimately suppressing lymphatic metastasis. Collectively, this work highlighted the therapeutic potential of CD169^+^ macrophage‐targeted nanomedicine and offered a promising framework for future precision immunotherapy against metastatic tumors.

## Conflict of Interest

The authors declare no conflict of interest.

## Supporting information



Supporting Information

## Data Availability

The data that support the findings of this study are available in the supplementary material of this article.
