# Supplementary material for: CD169+ Macrophage‐Targeted Immunomodulator to Restore Phagocytic Function and Enhance Antigen Presentation for Lymphatic Metastasis Eradication
Source: Adv Sci (Weinh). 2025 Nov 16;13(6):e14386. doi: 10.1002/advs.202514386 (PMC12866800; doi:10.1002/advs.202514386)
Supplement: Supplementary file 1 — Supporting Information [file ADVS-13-e14386-s001.docx]

Supporting Information

**CD169^+^ Macrophage-Targeted Immunomodulator to Restore Phagocytic Function and Enhance Antigen Presentation for Lymphatic Metastasis Eradication**

*Xiayun Chen, Lichong Lu, Yibin Liu, Ziqi Liang, Jianqiao Li, Zhouchuan Shao, Youzhi Tang, Jianhua Zou*, Shiying Li*, and Xiaoyuan Chen**

**Experimental Section**

**Materials**

1,2-Distearoyl-sn-glycero-3-phosphorylcholine (DSPC), cholesterol, 1,2-Distearoyl-sn-glycero-3-phosphorylethanolamine (DSPE), SHP099, and DMXAA were purchased from MedChemExpress. Ganglioside GM1 was acquired from RHAWN. Cy5.5 was sourced from Guyu. 3-(4,5-Dimethylthiazol-2-yl)-2,5-diphenyltetrazolium bromide (MTT), Calcein AM/PI staining kit and SDS-PAGE Gel preparation kit were provided by Beyotime Biotech. Dulbecco’s modified Eagle medium (DMEM), roswell park memorial institute (RPMI)-1640 medium, fetal bovine serum (FBS), and penicillin-streptomycin solution (PS) were obtained from Gibco. Antibodies against STING, pSTING, pTBK1, pIRF3, SHP2, GAPDH, and β-actin were supplied by Abcam. Antibodies against CD45-percp-cy5.5, CD3-APC-cy7, CD4-FITC, CD8-PE, CD11C-FITC, CD80-BV421, CD86-PE, CD11b-APC, Ly6c-FITC, IFNγ-APC, MHCII-APC, Foxp3-PE and TIM-3-APC were provided by BD Biosciences. IFN-α and M-CSF cytokines were purchased from Proteintech. Cytokine detection kits for IFN-α, IFN-β, IFN-γ, CXCL9, and CXCL10 were obtained from Xinbosheng.

**Cell Culture Medium**

4T1, 4T1-GFP and RAW264.7 cells were cultured in DMEM supplemented with 10 % FBS and 1% penicillin streptomycin (PS, penicillin and streptomycin). CTLL-2 cells were cultured in DMEM supplemented with 10 % FBS, 1 % PS and 0.1 ‰ IL-2. Cells were maintained in a humidified incubator at 37 °C with 5 % CO_2_ to ensure optimal growth conditions. CD169^+^ macrophages were derived from primary cells isolated from the bone marrow of BALB/c mice and stimulated for differentiation. Specifically, bone marrow cells (1.5-2×10^7^) from BALB/c mice were collected and differentiated into macrophages *in vitro* using RPMI-1640 medium containing 10 % FBS, 1 % PS, and 10 ng/mL M-CSF. On day 7, IFN-α (500 IU/mL) was added to the medium for 2 days to induce CD169 expression.

**Bioinformatics Analysis and Experimental Validation *in Vitro***

The correlation between CD169 and CD4, CD8, and SHP2 was analyzed using the TIMER database (https://cistrome.shinyapps.io/timer/). CD169^+^ macrophages were co-cultured with or without 4T1 cells in transwell plates for 24 hours, and then co-incubated with 4T1-GFP cells for another 2 hours. Following incubation, the cells were collected for flow cytometry analysis to assess the phagocytosis rate of CD169^+^ macrophages. Additionally, CD169^+^ macrophages were collected for flow cytometry analysis to examine the expression of CD80, CD86, MHCI, and MHCII after co-culturing with or without 4T1 cells in transwell plates for 24 hours.

**Synthesis and Characterization of G-LNP@S-D**

*Preparation of G-LNP@S-D and G-LNP*: DSPC, cholesterol, DSPE, GM1, SHP099, and DMXAA were mixed in a molar ratio of 55:35:5:5:5.8:26 and stirred for 10 minutes. After that, the solution was collected and subjected to rotary evaporation to remove volatile liquids. Following rotary evaporation, 1 mL ultrapure water was added, and the lipid membrane was hydrated by sonication for 10 minutes. The solution was then centrifuged at 2000 rpm for 5 minutes to remove unincorporated free drugs, and G-LNP@S-D was obtained by collecting the supernatant. The same steps were repeated after mixing lipids DSPC, cholesterol, DSPE, and GM1 in a molar ratio of 55:35:5:5 to obtain G-LNP. To prepare fluorescently labeled liposomes, 1 % cy5.5 was added to the liposomes.

*Characterizations of G-LNP@S-D and G-LNP:* G-LNP@S-D or G-LNP with different feed ratios were negatively stained with 1% phosphotungstic acid and their morphology was observed under TEM. The particle size and zeta potential of G-LNP@S-D and G-LNP were measured using a dynamic light scattering analyzer, and the hydrated particle size and PDI values were continuously monitored and recorded to assess their stability in aqueous solution.

*Drug Loading of G-LNP@S-D:* The drug loading of SHP099 and DMXAA in G-LNP@S-D was determined using high-performance liquid chromatography (HPLC). SHP099 standard solutions with concentrations of 1, 2, 4, 8, 16, 32, and 64 mg/L and DMXAA standard solutions with concentrations of 5, 10, 20, 40, 60, 80, and 100 mg/L were prepared. The chromatographic peak areas of the G-LNP@S-D sample solution were then substituted into standard equations to calculate the drug content of SHP099 and DMXAA in G-LNP@S-D.

**Cellular Uptake Behavior of G-LNP@S-D**

*Cellular Uptake in Different Cells*: Cy5.5-labeled G-LNP@S-D was prepared. 4T1 cells, macrophages (RAW264.7), and CD169^+^ macrophages were incubated with G-LNP@S-D in different concentrations (1, 3, and 5 mg/L) for 4 hours. After incubation, the cells were stained with the nuclear dye Hoechst 33342 for 15 minutes. Then intracellular cy5.5 fluorescence was observed and quantitatively analyzed by confocal laser scanning microscope (CLSM).

*GM1 Mediated Cellular Uptake and DMXAA Mediated Uptake Upregulation:* Cy5.5-labeled G-LNP@S, G-LNP@D, LNP@S-D, G-LNP@S-D and anti-CD169 + G-LNP@S-D were prepared and added (containing 3 mg/L of DMXAA) into CD169^+^ macrophages to co-incubate for 4 hours. After that, cy5.5 fluorescence was quantitatively analyzed using flow cytometer.

*Western Blotting Detection of CD169 Expression:* CD169^+^ macrophages were treated with SHP099 (2.0 mg/L), DMXAA (6.6 mg/L), or G-LNP@S-D (68.6 mg/L) for 48 hours. Then the cells were collected for western blot analysis to detect the expression of CD169. Grayscale values were statistically analyzed using ImageJ.

**Phagocytosis Restoration of CD169^+^ Macrophages Mediated by G-LNP@S-D**

*MTT Assay*: 4T1 cells or CD169^+^ macrophages were cultured with SHP099 (5 mg/L), DMXAA (16.5 mg/L), or G-LNP@S-D (171.5 mg/L) for 24 hours. After 24 hours, 20 µL MTT solution was added and further incubated for 4 hours. Following this, the solution was replaced by 150 µL DMSO and the cells were shaken for 15 minutes. The absorbance at 570 nm was measured by microplate reader.

*Western Blot to Detect SHP2 Expression*: CD169^+^ macrophages were incubated with SHP099 (5.0 mg/L), DMXAA (16.5 mg/L), or G-LNP@S-D (171.5 mg/L) for 48 hours. The cells were then lysed, and proteins were collected to detect SHP2 expression and grayscale values were statistically analyzed using Image J.

*Phagocytosis Assay*: CD169^+^ macrophages were treated with SHP099 (5.0 mg/L), DMXAA (16.5 mg/L), or G-LNP@S-D (171.5 mg/L) for 24 hours. Subsequently, the CD169^+^ macrophages were co-cultured with 4T1-GFP cells. After 2 hours, the cells were collected and stained with PB450-labelled CD80 for 30 minutes. The double-positive rate of the two fluorescence was measured and statistically analyzed using flow cytometer. Additionally, The GFP fluorescence of 4T1 cells after the same operation was monitored throughout the real-time microscope.

*Transwell Experiment:* CD169^+^ macrophages were seeded in the upper chamber of a transwell plate and treated with SHP099 (5.0 mg/L), DMXAA (16.5 mg/L), or G-LNP@S-D (171.5 mg/L) for 24 hours. Afterward, 4T1 cells were seeded in the lower chamber for 24 hours of co-incubation. Following this, CTLL-2 cells were added and co-cultured for another 24 hours. Apoptosis detection kits were used to investigate the apoptosis of 4T1 cells and the anti-apoptotic ability of CTLL-2 cells. Additionally, TIM-3 on CTLL-2 cells was detected by flow cytometry.

**STING Activation and Enhanced Antigen Presentation of CD169^+^ Macrophages Induced by G-LNP@S-D**

*STING Activation*: CD169^+^ macrophages were incubated with SHP099 (2.0 mg/L), DMXAA (6.6 mg/L), or G-LNP@S-D (68.6 mg/L) for 42 hours. The cells were then lysed, and collected to detect the expression of STING, pSTING, pIRF3, and pTBK1, followed by grayscale analysis using ImageJ.

*Cytokine Detection:* CD169^+^ macrophages were treated with SHP099 (5.0 mg/L), DMXAA (16.5 mg/L), or G-LNP@S-D (171.5 mg/L) for 24 hours. Subsequently, the cell supernatants were collected and ELISA kits were used to detect the release of IFN-α, IFN-β, IFN-γ, CXCL9, and CXCL10.

*Detection of Surface Marker Proteins*: CD169^+^ macrophages were treated with SHP099 (5.0 mg/L), DMXAA (16.5 mg/L), or G-LNP@S-D (171.5 mg/L) for 24 hours. Subsequently, 4T1 cells were added to culture for another 24 hours. The expression of MHCI, MHCII, and CD80^+^CD86^+^ on CD169^+^ macrophages was detected by flow cytometry.

**Biodistribution of G-LNP@S-D and LNP@S-D**

All animal experiments were conducted in accordance with the Regulations on the Administration of Laboratory Animal Affairs and approved by the Institutional Animal Care and Use Committee of the Animal Experiment Center of Guangzhou Medical University (Approval number: GY2023-711). A mouse model of lymph node metastatic tumor was established by injecting a suspension of 4T1 cells (1×10^5^) into the footpad of BALB/c female mouse (6-7 weeks old). Cy5.5-labeled G-LNP@S-D and LNP@S-D were then administered *via* subcutaneous injection. Fluorescence accumulation in the primary tumor at the footpad and popliteal lymph nodes (LNs) was observed at 0, 2, 6, 12, 24, 36 and 48 hours post-injection. Following imaging, tumors, popliteal LNs, as well as the heart, liver, spleen, lung, and kidney tissues of the mice were collected for *in vitro* fluorescence imaging and photography. The tumors and bilateral popliteal LNs of the mice were subjected to fluorescence staining to observe the colocalization of G-LNP@S-D with CD169^+^ macrophages.

**Primary and Lymph Node Metastatic Tumors Inhibition by G-LNP@S-D**

4T1 cells were digested, washed three times with PBS, and resuspended in PBS. 50 µL single-cell suspension (1×10^5^ cells) was injected into the left footpad of BALB/c female mice (6-7 weeks old) using a syringe to establish a mouse model of 4T1 lymph node metastatic tumor. Nine days later, the mice were treated with subcutaneous injections of saline, SHP099 (7.5 mg/kg), DMXAA (25.0 mg/kg), SHP099 + DMXAA (32.5 mg/kg), or G-LNP@S-D (257.3 mg/kg). Treatments were administered every other day for a total of three treatments over an 11-day period. During this time, the tumor volume and body weight of the mice were recorded every other day. On the 12^th^ day, the tumors at the footpad, popliteal LNs, and spleens of the mice were collected for photography and weighing. The organs and tumors were subjected to H&E staining and TUNEL staining.

**Immune Cell Analysis**

4T1 cells were digested, washed three times with PBS, and resuspended in PBS. 50 µL single-cell suspension (1×10^5^ cells) was injected into the left footpad of BALB/c female mice (6-7 weeks old) using a syringe to establish a mouse model of 4T1 lymph node metastatic tumor. Nine days later, the mice were treated with subcutaneous injections of saline, SHP099 (7.5 mg/kg), DMXAA (25.0 mg/kg), SHP099 + DMXAA (32.5 mg/kg), or G-LNP@S-D (257.3 mg/kg). Treatments were administered every other day for a total of three treatments. Following the third treatment, the mice were euthanized by cervical dislocation, and tumors, popliteal LNs, and spleen tissues were collected. These tissues were homogenized into cell suspensions, stained, and analyzed for relevant immune cells using flow cytometry. Additionally, popliteal LNs from the mice were subjected to fluorescent staining for CD169, CD4, or CD8, and the fluorescence intensity was quantitatively analyzed.

**Biosafety Analysis**

After the treatment period, blood samples were collected from the mice *via* venipuncture for biochemical analysis. The hearts, livers, and kidneys of the mice were collected and subjected to H&E staining to characterize the morphology of these organs.

**Statistical Analysis**

For the comparison between two sets of data, Student's t-test was used. One-way ANOVA analysis was employed among multiple groups comparisons. Statistical significance was indicated as **P* < 0.05, ***P* < 0.01, ****P* < 0.001.


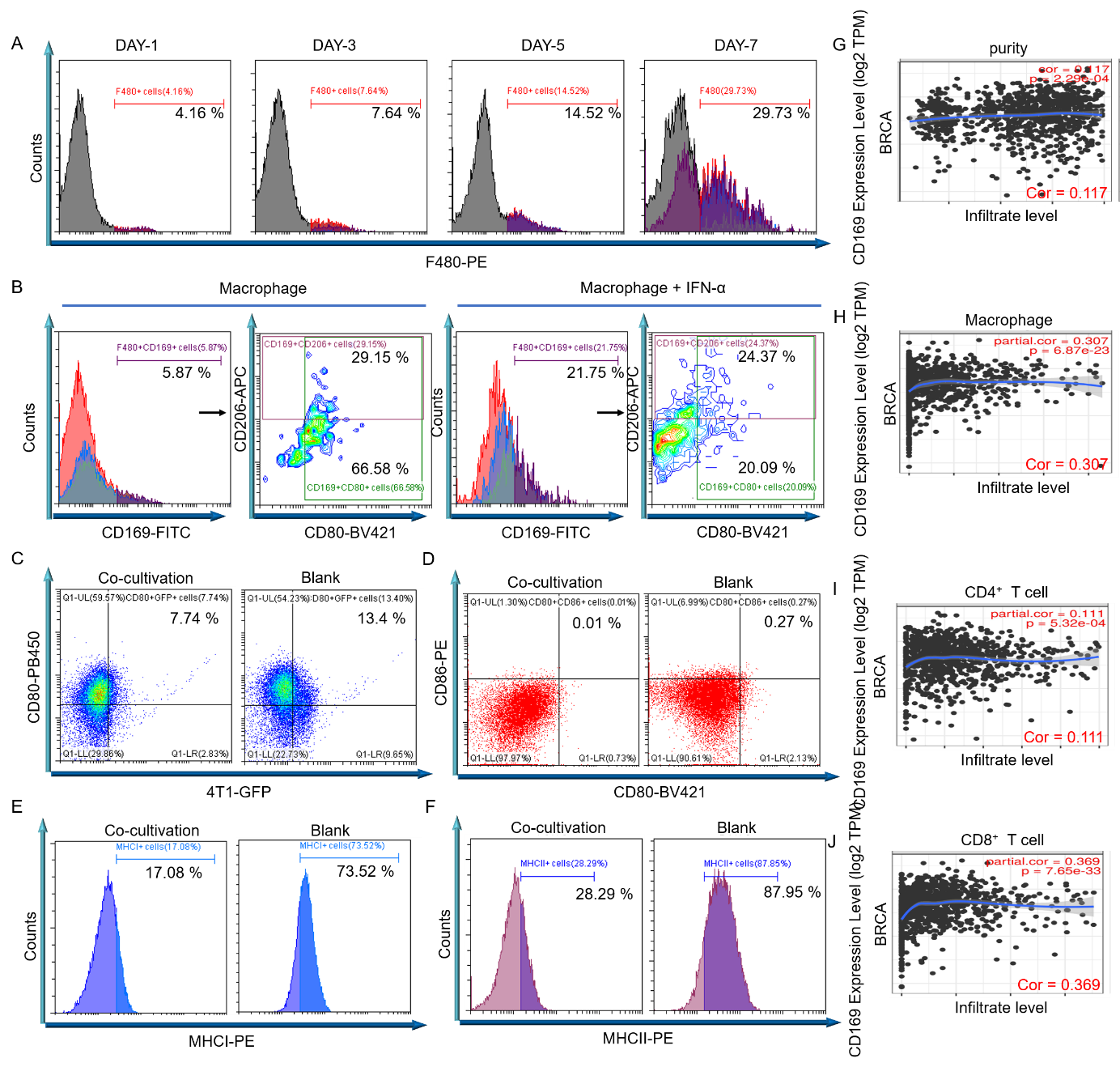


**Figure S1.** (A) The flow cytometry analysis of macrophages in bone marrow monocytes induced by M-CSF on the first, third, fifth, and seventh days. (B) The flow cytometry analysis of CD169^+^ cells in macrophages with or without IFN-α induction, as well as flow cytometry analysis of CD169^+^CD80^+^ cells and CD169^+^CD206^+^ cells. Representative flow analysis chart of (C) CD80^+^GFP^+^, (D) CD80^+^CD86^+^, (E) MHCI^+^, (F) MHCII^+^ macrophages in co-cultivation group (4T1 cells + CD169 macrophages) or Blank group (only CD169 macrophages). Correlation between CD169 expression and (G) tumor purity, (H) macrophage, (I) CD4^+^ T cell or (J) CD8^+^ T cell in tumor tissues analyzed *via* the TIMER database.


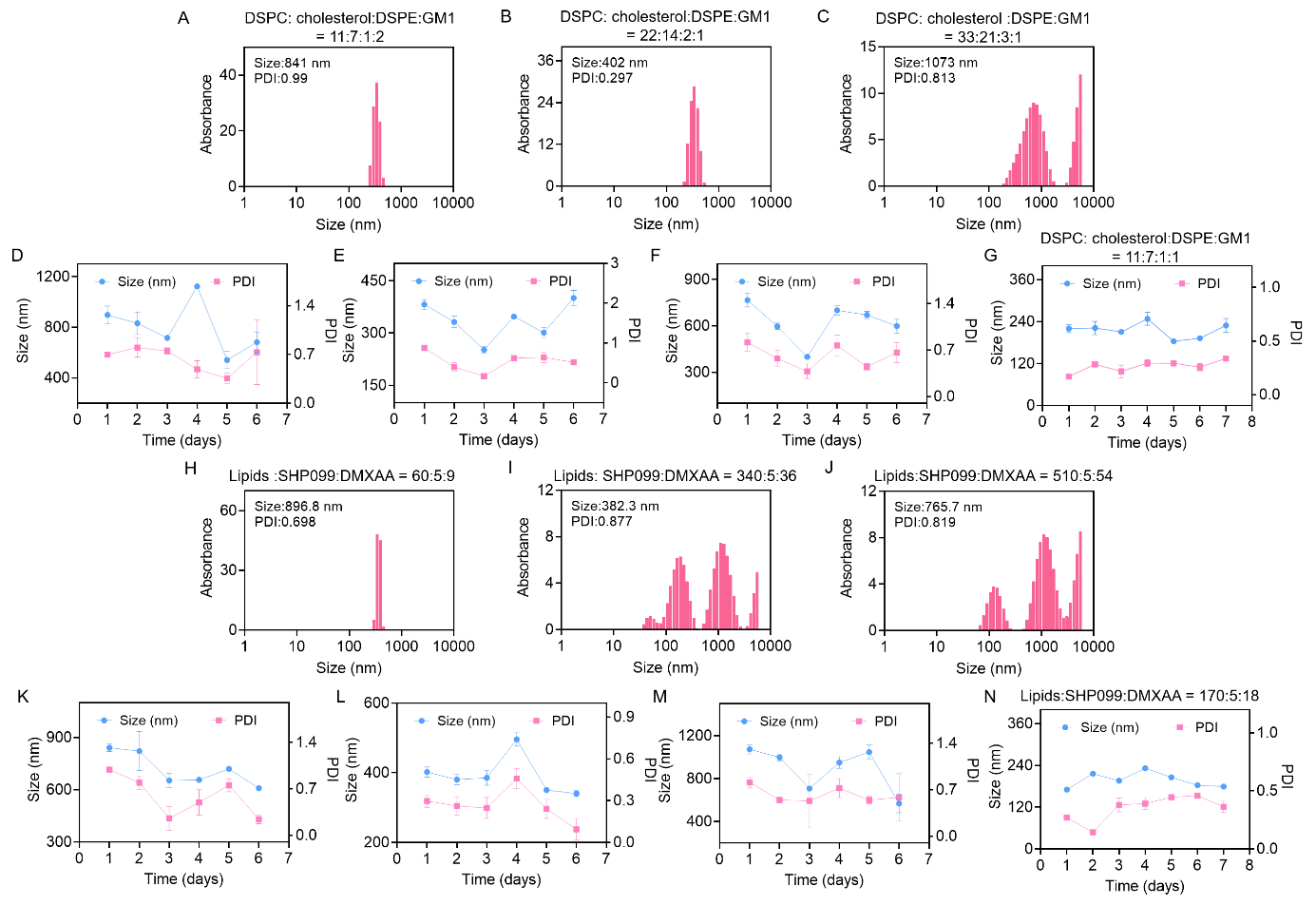


**Figure S2**. Particle size distributions obtained based on the assembly of DSPC, cholesterol, DSPE and GM1 at the ratios of (A) 11:7:1:2, (B) 22:14:2:1 or (C) 33:21:3:1 and (D-G) the changes of particle size and PDI within a certain period of time. Particle size distributions obtained based on the assembly of lipids, SHP099 and DMXAA at the ratios of (H) 60:5:9, (I) 340:5:36 or (J) 510:5:54 and (K-N) the changes of particle size and PDI within a certain period of time.


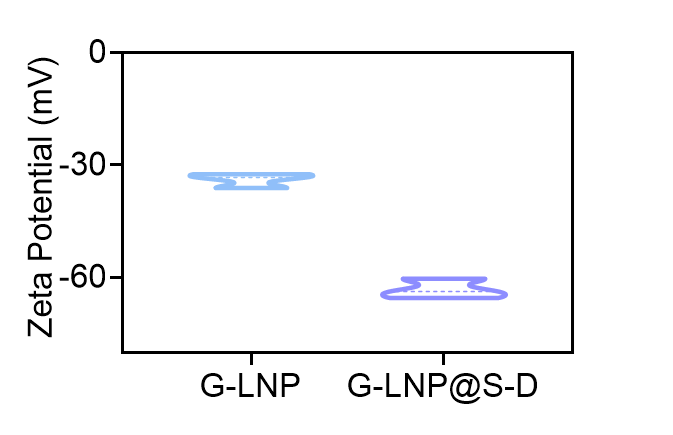


**Figure S3**. ζ-potential of G-LNP and G-LNP@S-D (n = 3).


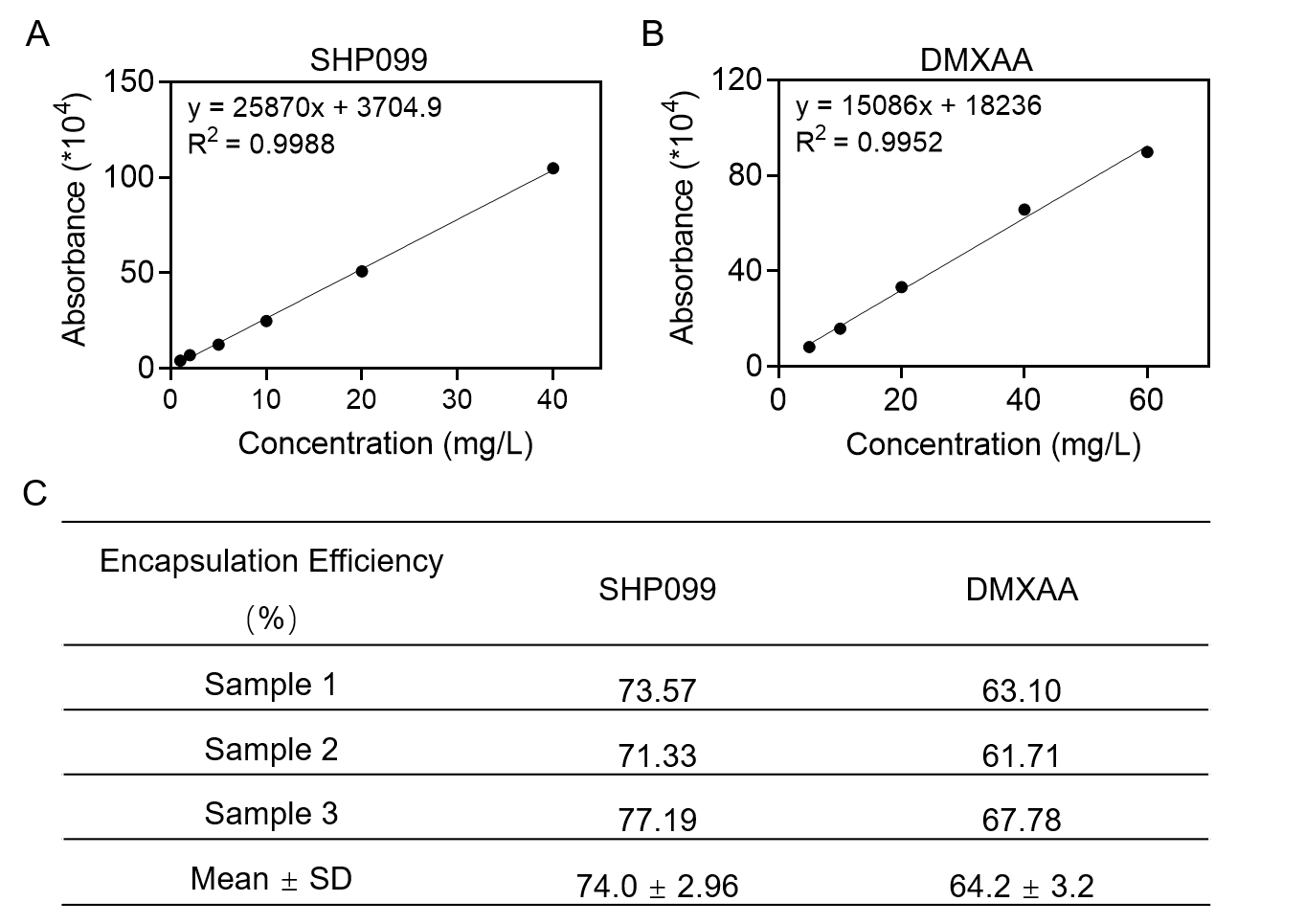


**Figure S4**. Concentration standard curve of (A) SHP099 and (B) DMXAA. (C) Encapsulation efficiency of three parallel samples of G-LNP@S-D.


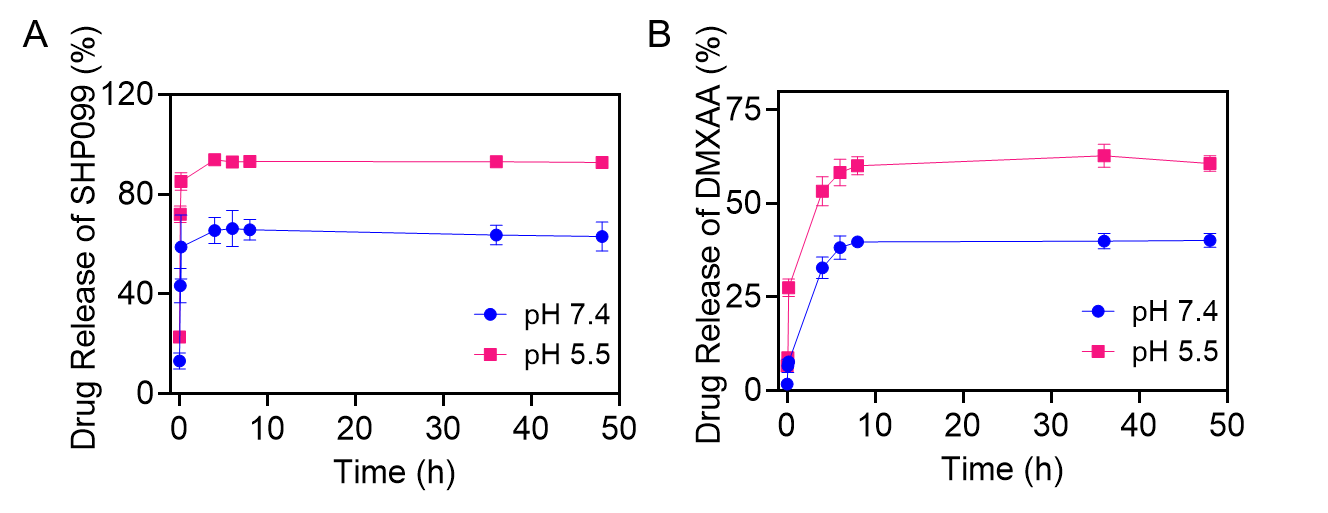


**Figure S5**. Drug release curve of (A) SHP099 and (B) DMXAA in G-LNP@S-D.


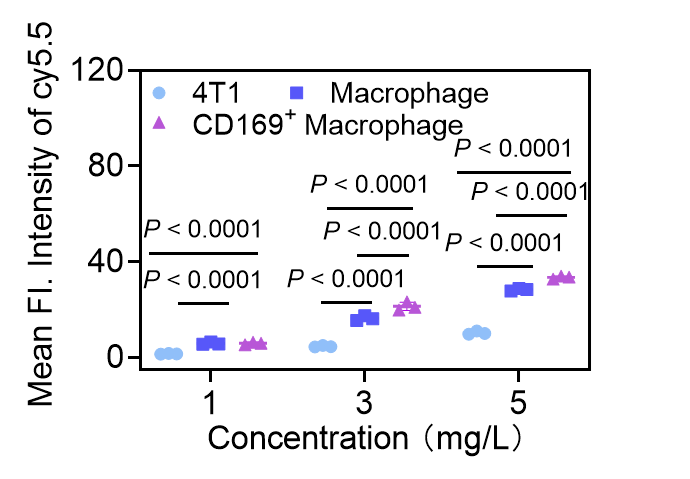


**Figure S6.** Corresponding quantification of 4T1 cells, RAW264.7 macrophages, and CD169^+^ macrophages incubated with Cy5.5-labeled G-LNP@S-D at varying concentrations (1, 3, 5 mg/L). (n = 3). P values were tested *via* a one-way ANOVA analysis.


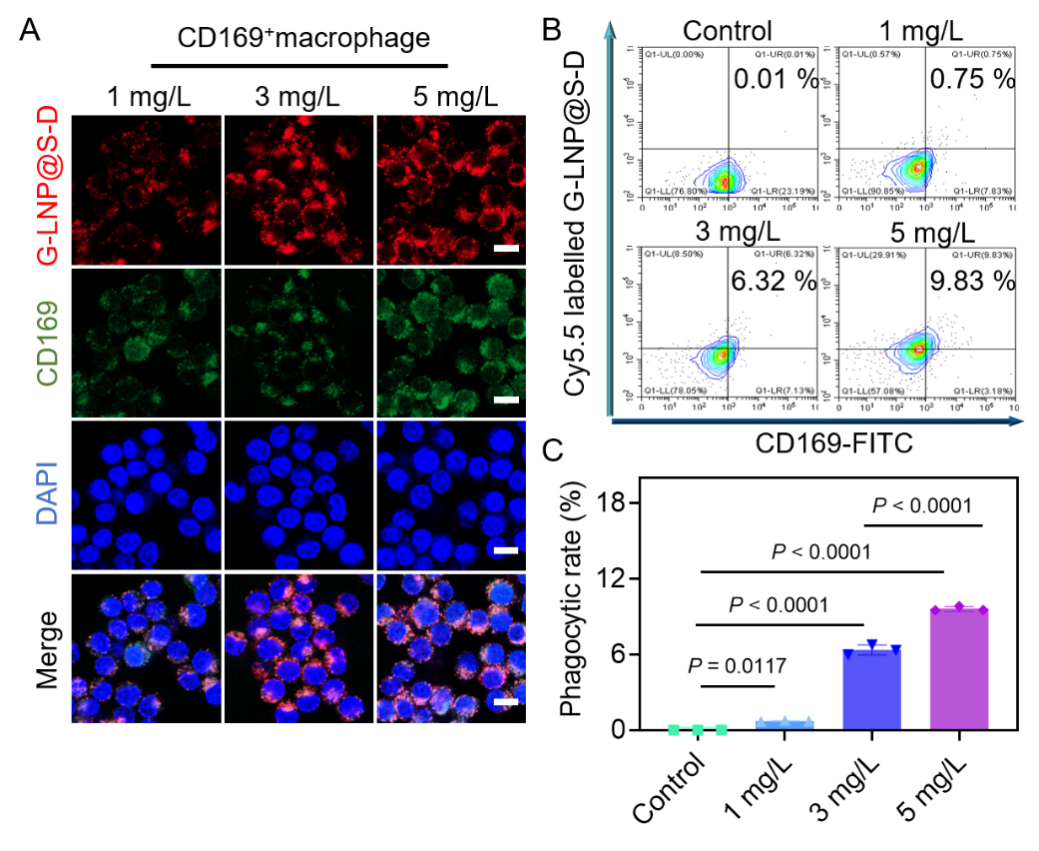


**Figure S7**. (A) CLSM images of CD169^+^ macrophages after treatment with different concentrations (1, 3, 5 mg/L) of cy5.5 labelled G-LNP@S-D and FITC labelled CD169 (n = 3). Scale bar: 10 µm. (B) Flow cytometry analysis and (C) statistical analysis of CD169^+^ macrophages after same treatments. P values were tested *via* a one-way ANOVA analysis.


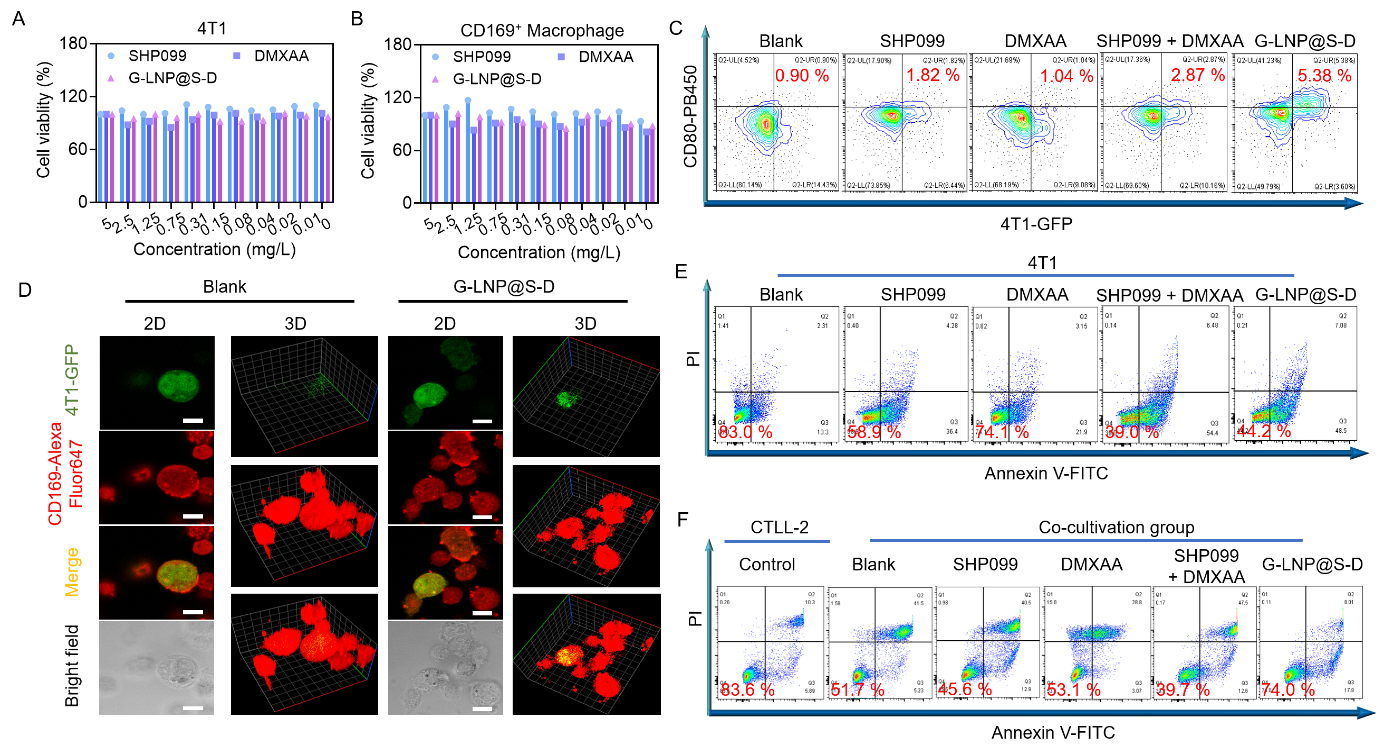


**Figure S8.** Cell viabilities of (A) 4T1 cells or (B) CD169^+^ macrophages after treatment with SHP099, DMXAA or G-LNP@S-D. (C) Flow cytometry analysis of the phagocytic elimination of tumor cells by CD169^+^ macrophages after treatment with SHP099, DMXAA, SHP099 + DMXAA or G-LNP@S-D. (D) CLSM observation of the phagocytic elimination behavior of CD169^+^ macrophages after treatment with PBS (Blank) or G-LNP@S-D. Scale bar: 10 µm. (E) Flow cytometry measurement of PI/Annexin V-FITC staining of 4T1 cells after treatment with SHP099, DMXAA, SHP099 + DMXAA or G-LNP@S-D. (F) Flow cytometry measurement of PI/Annexin V-FITC staining of CTLL-2 cells after treatment with SHP099, DMXAA, SHP099 + DMXAA or G-LNP@S-D.


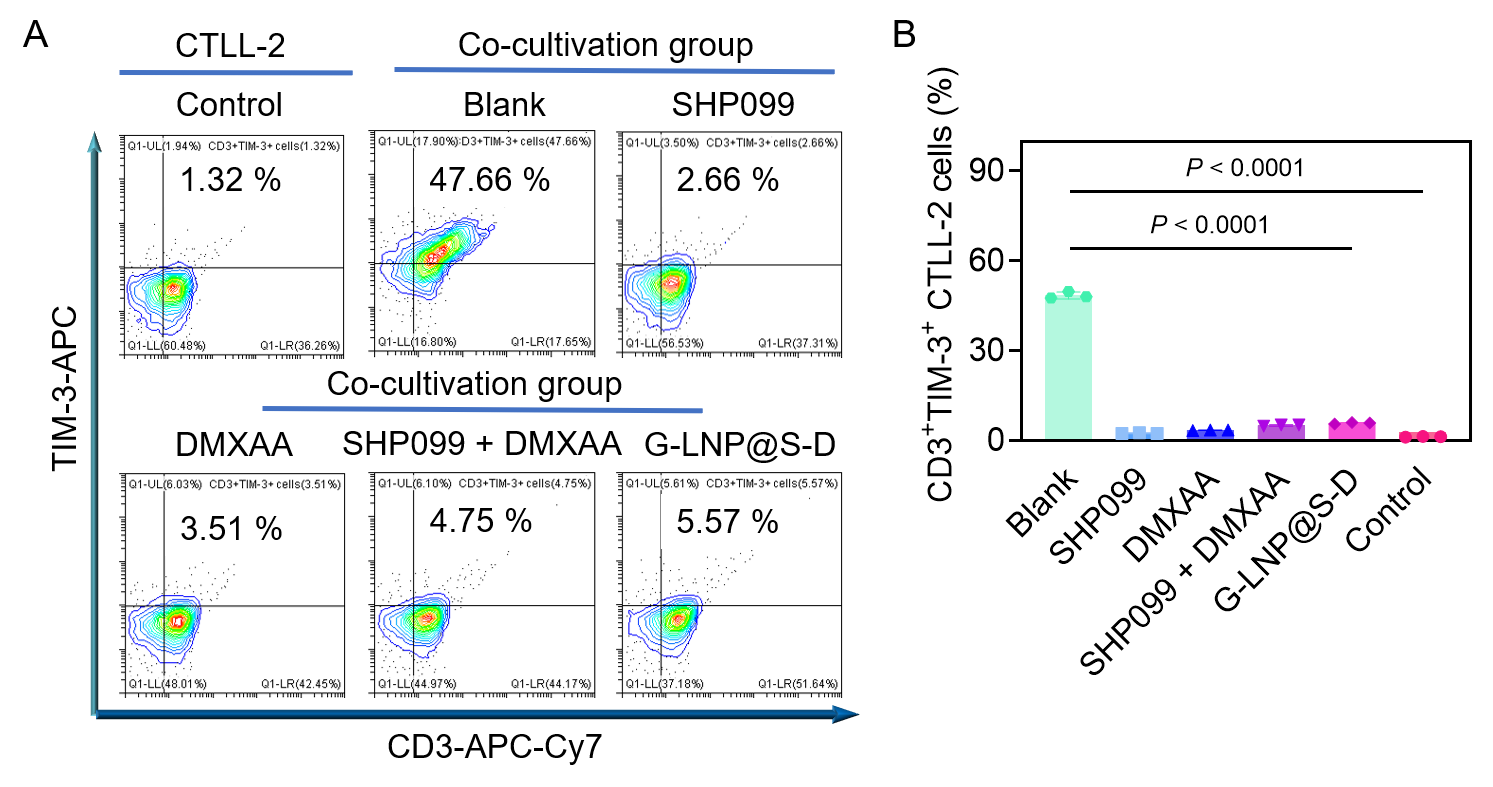


**Figure S9**. (A) Flow cytometry analysis and (B) statistical analysis of exhaustion of T cells after co-incubation with CD169^+^ macrophages treated with SHP099, DMXAA, SHP099 + DMXAA or G-LNP@S-D and 4T1 cells. P values were tested *via* a one-way ANOVA analysis.


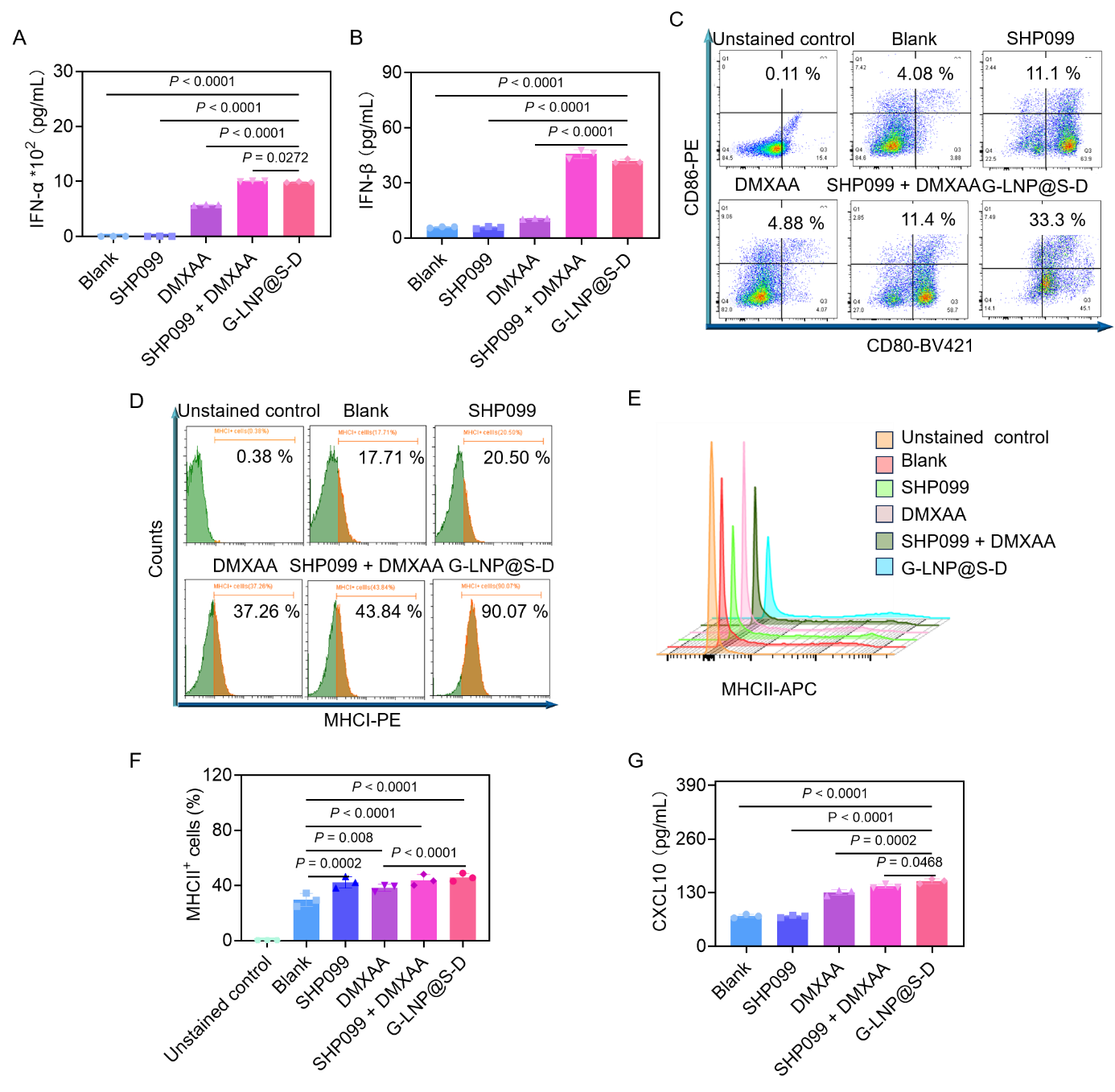


**Figure S10**. The expression of (A) IFN-α, and (B) IFN-β after treatment with SHP099, DMXAA, SHP099 + DMXAA or G-LNP@S-D. Representative flow analysis chart of (C) CD80^+^CD86^+^, (D) MHCI^+^, or (E) MHCII^+^ on CD169^+^ macrophages after treatment with SHP099, DMXAA, SHP099 + DMXAA or G-LNP@S-D. (F) Corresponding data analysis of MHCI^+^ cells after same treatments. (G) CXCL10 expression on CD169^+^ macrophages after treatment with SHP099, DMXAA, SHP099 + DMXAA or G-LNP@S-D. P values were tested *via* a one-way ANOVA analysis.


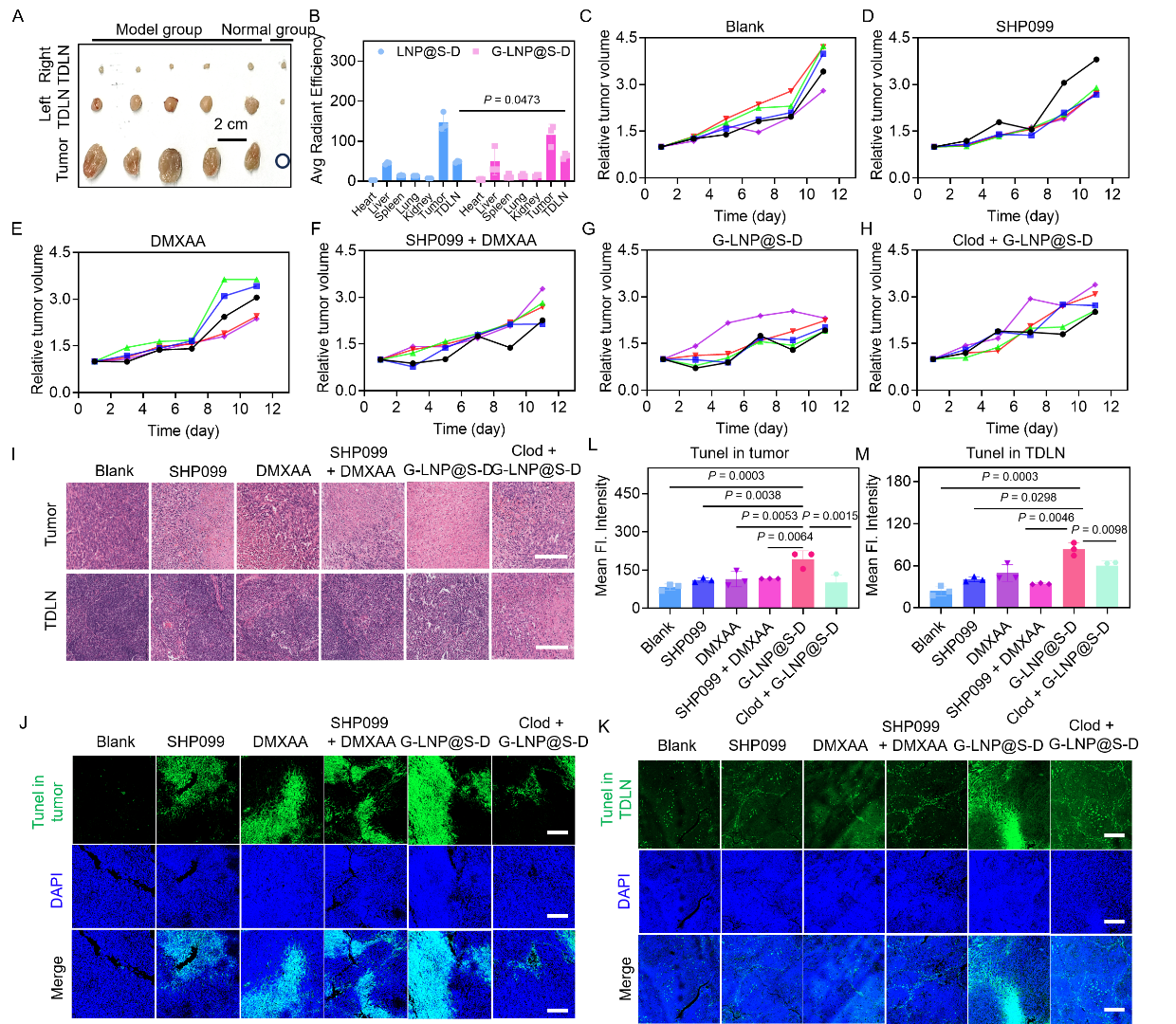


**Figure S11**. (A) Representative images of tumor-draining lymph nodes (TDLNs) and primary tumors in 4T1-bearing versus healthy mice. (B) Quantitative fluorescence intensity of different tissues 48 h post subcutaneous injection with LNP@S-D or G-LNP@S-D. Tumor volume changes of mice after treatment with (C) PBS, (D) SHP099, (E) DMXAA, (F) SHP099 + DMXAA, (G) G-LNP@S-D or (H) Clod + G-LNP@S-D in 11 days. (I) H&E staining and representative images of tumor and TDLN after treatment with SHP099, DMXAA, SHP099 + DMXAA, G-LNP@S-D or Clod + G-LNP@S-D on the 11th day. Scale bar: 200 µm. TUNEL staining of (J) tumor or (K) TDLN after treatment with SHP099, DMXAA, SHP099 + DMXAA, G-LNP@S-D or Clod + G-LNP@S-D. The corresponding statistical analysis of TUNEL staining of (L) tumor or (M) TDLN after same treatments. All the tissue sections were counterstained with DAPI (blue). Scale bar: 100 µm. P values were tested *via* a one-way ANOVA analysis.


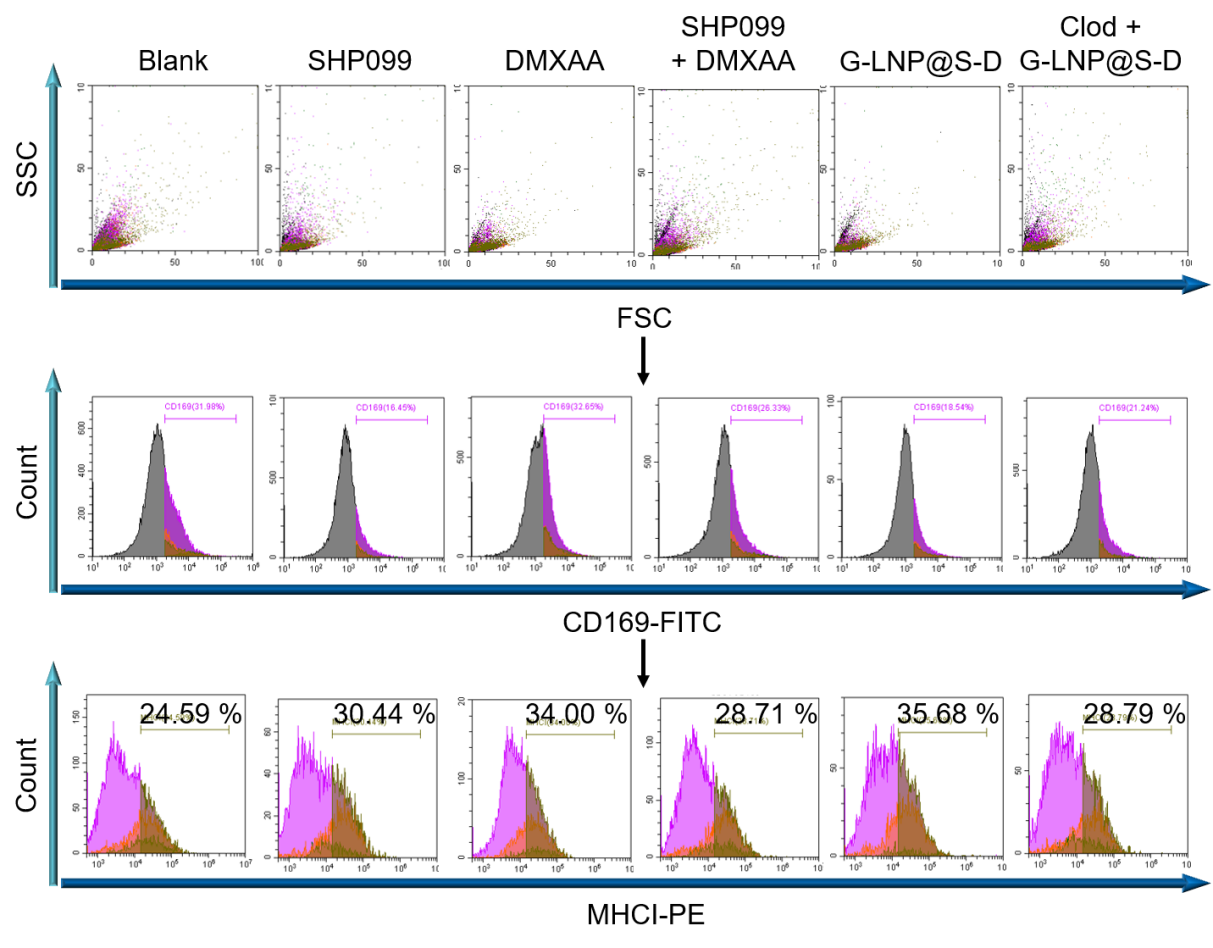


**Figure S12**. Representative flow analysis chart of CD169^+^MHCI^+^ macrophages in TDLN after treatment with SHP099, DMXAA, SHP099 + DMXAA, G-LNP@S-D or Clod + G-LNP@S-D.


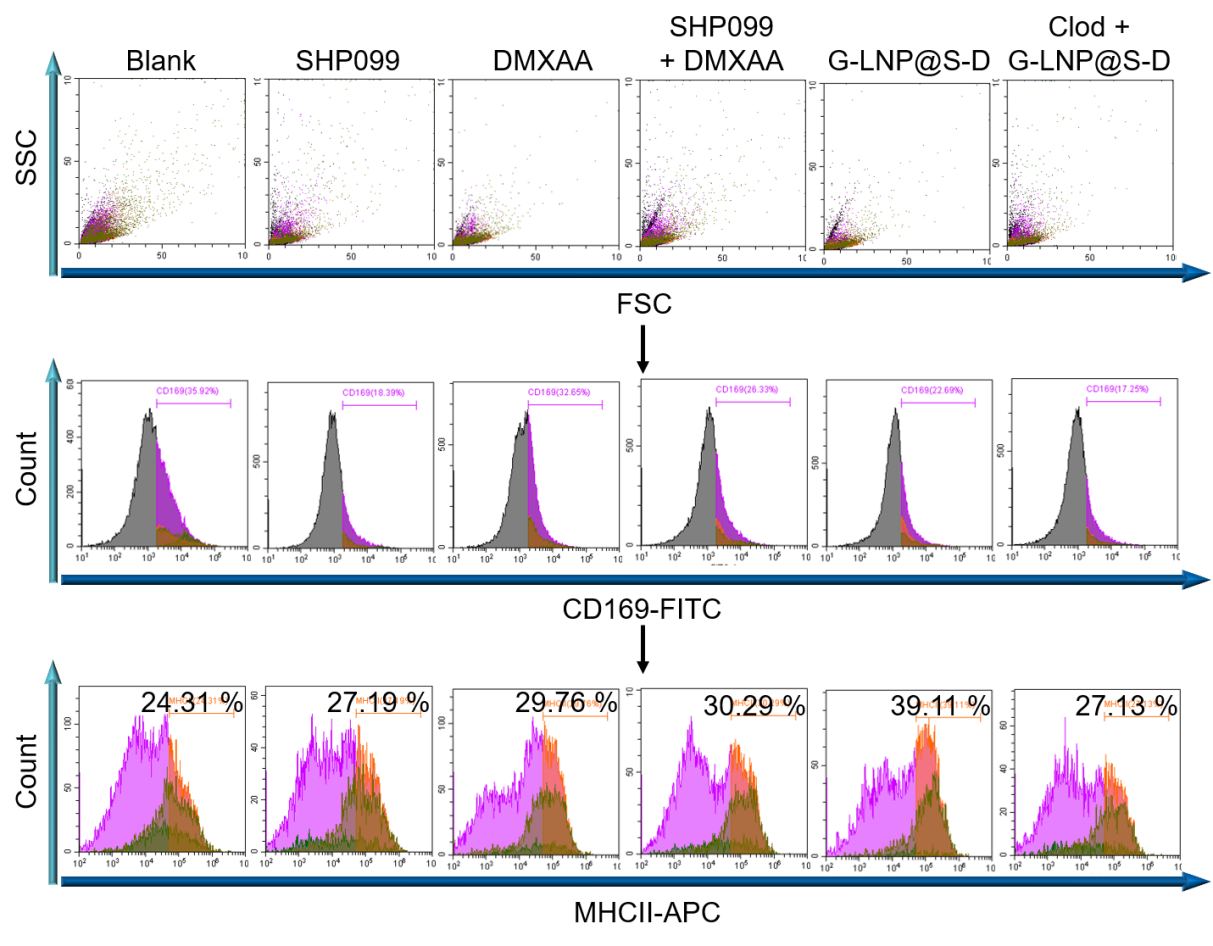


**Figure S13**. Representative flow analysis chart of CD169^+^MHCII^+^ macrophages in TDLN after treatment with SHP099, DMXAA, SHP099 + DMXAA, G-LNP@S-D or Clod + G-LNP@S-D.


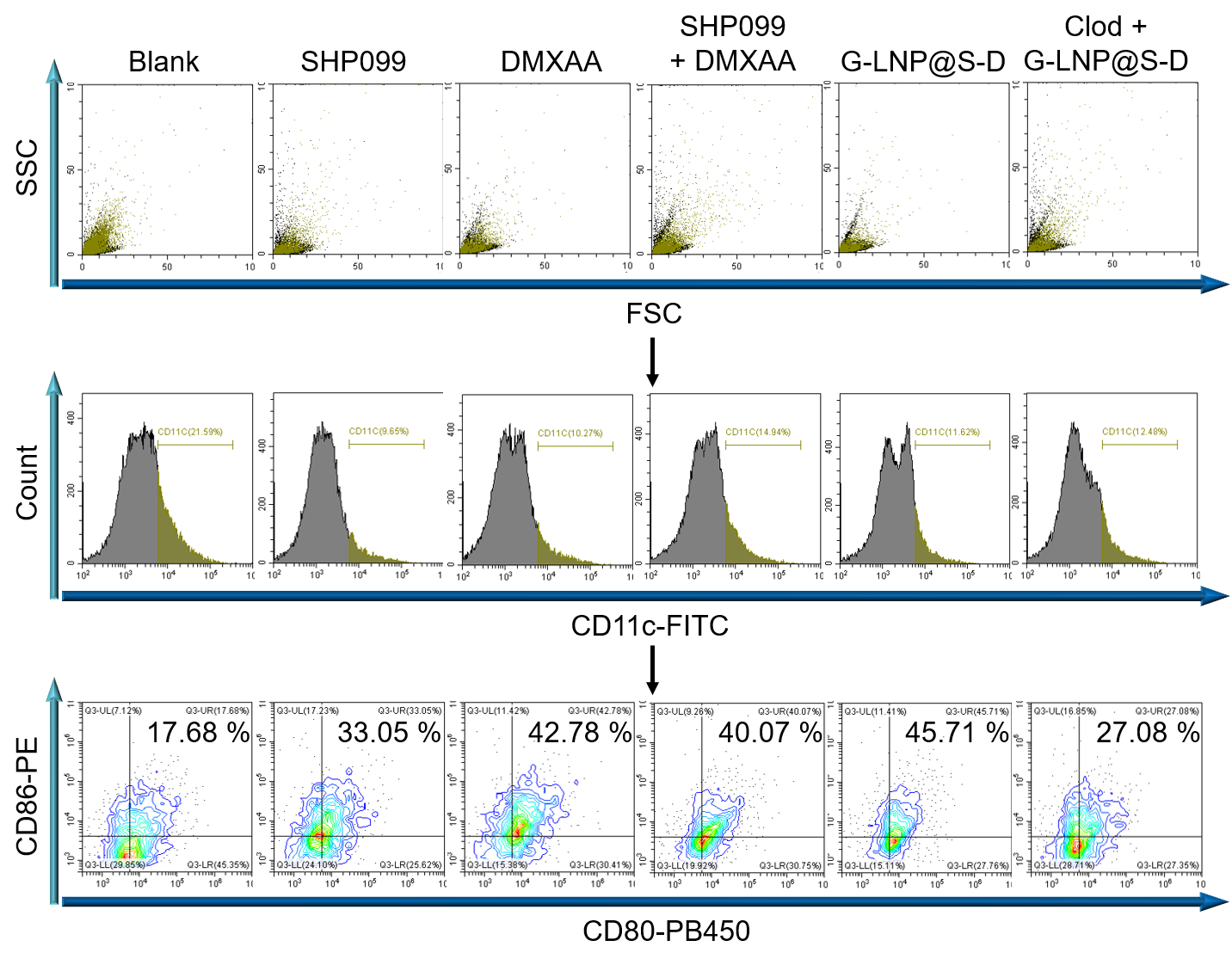


**Figure S14**. Representative flow analysis chart of CD80^+^CD86^+^ macrophages in TDLN after treatment with SHP099, DMXAA, SHP099 + DMXAA, G-LNP@S-D or Clod + G-LNP@S-D.


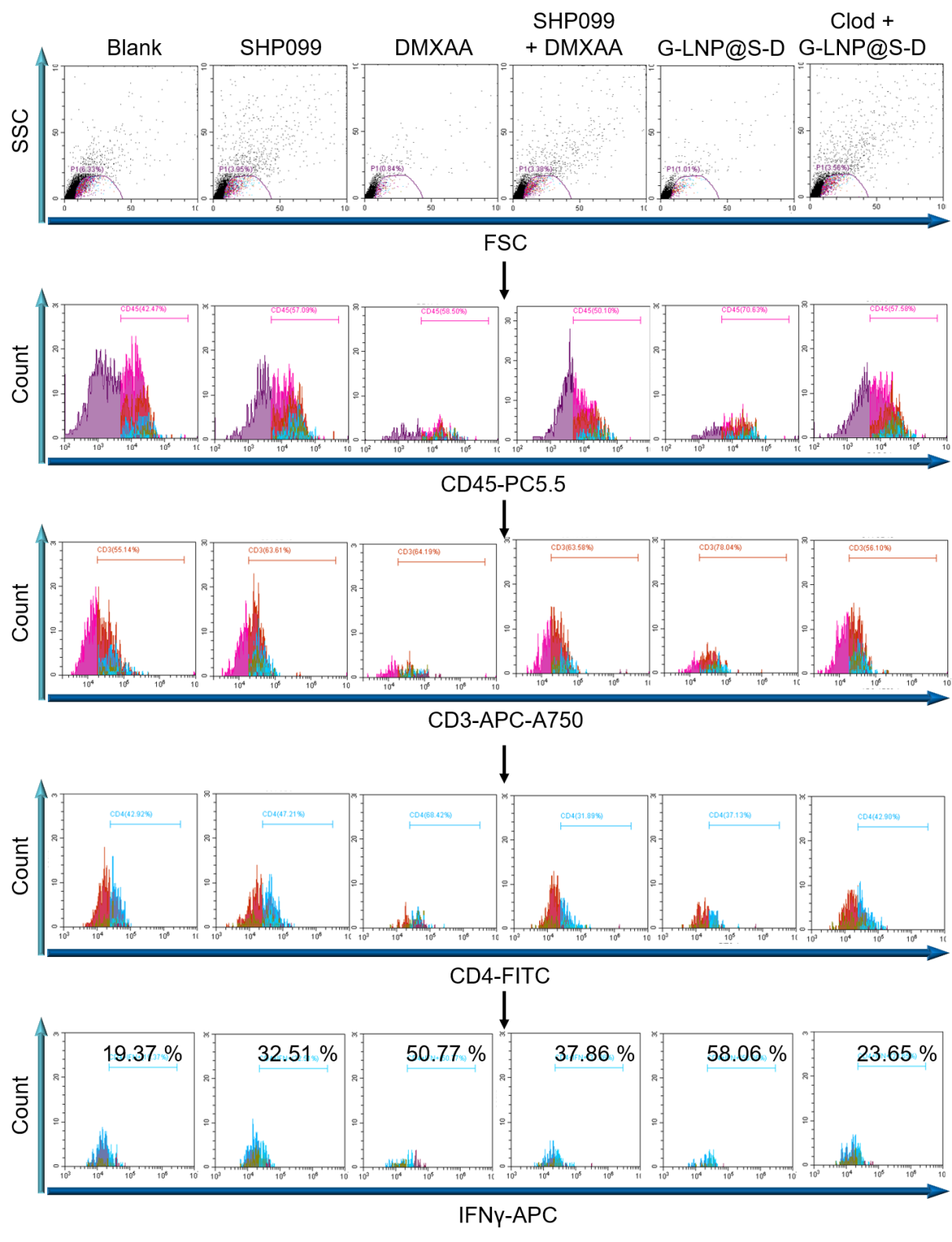


**Figure S15**. Representative flow analysis chart of CD4^+^IFNγ^+^ T lymphocytes in TDLN after treatment with SHP099, DMXAA, SHP099 + DMXAA, G-LNP@S-D or Clod + G-LNP@S-D.


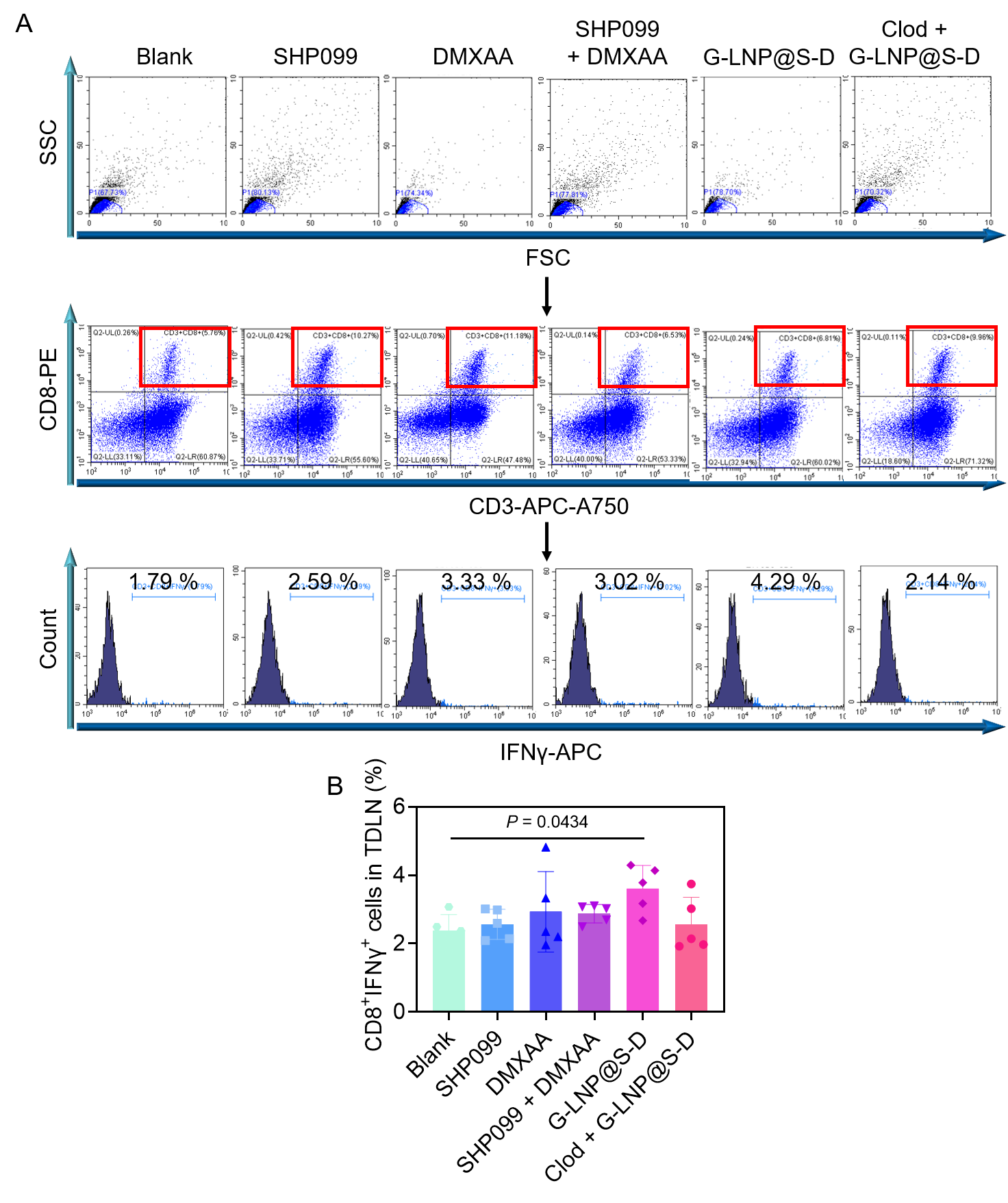


**Figure S16.** (A) Representative flow analysis chart and (B) corresponding quantitative analysis of CD8^+^IFNγ^+^ T lymphocytes in TDLN after treatment with SHP099, DMXAA, SHP099 + DMXAA, G-LNP@S-D or Clod + G-LNP@S-D. P values were tested *via* a one-way ANOVA analysis.


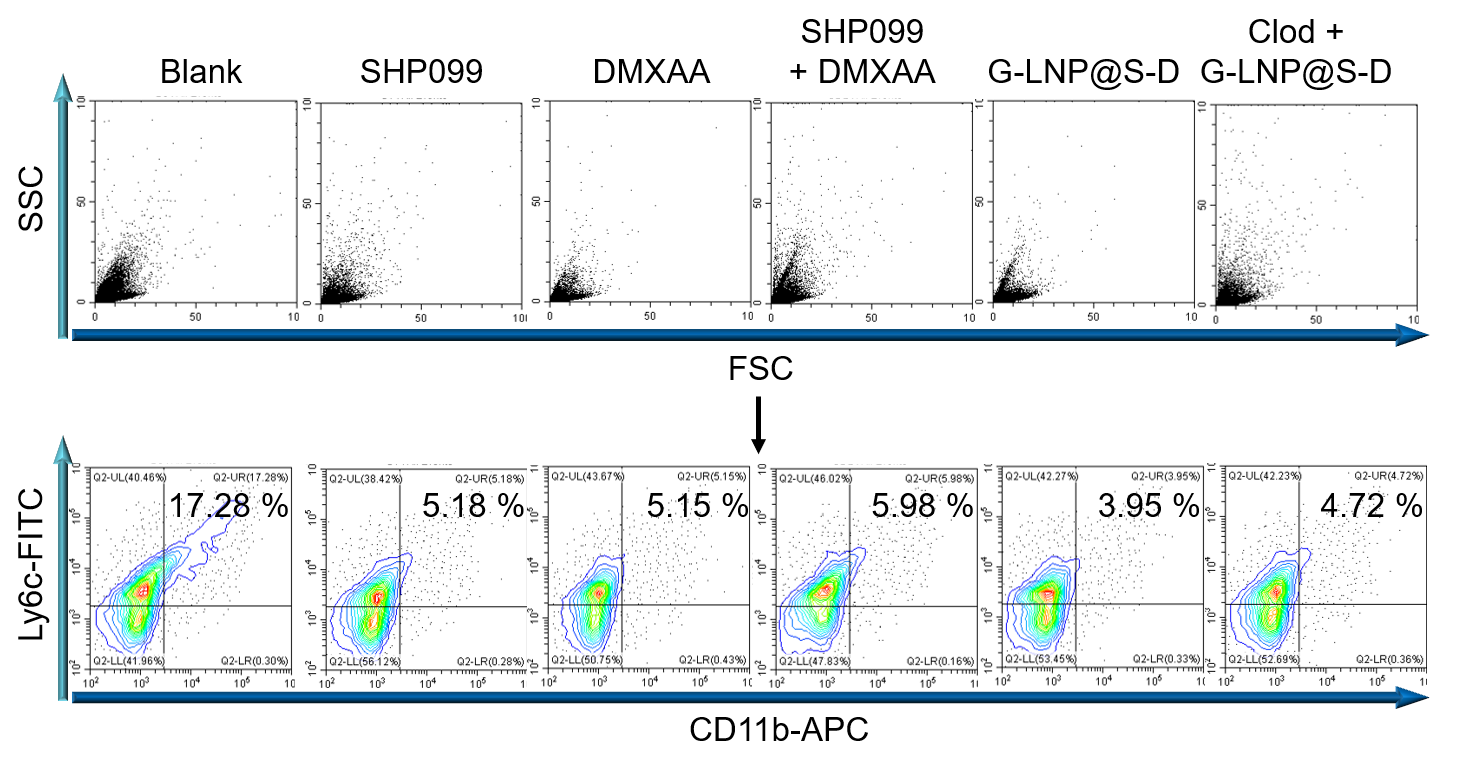


**Figure S17.** Representative flow analysis chart of CD11b^+^Ly6c^+^ cells in TDLN after treatment with SHP099, DMXAA, SHP099 + DMXAA, G-LNP@S-D or Clod + G-LNP@S-D.


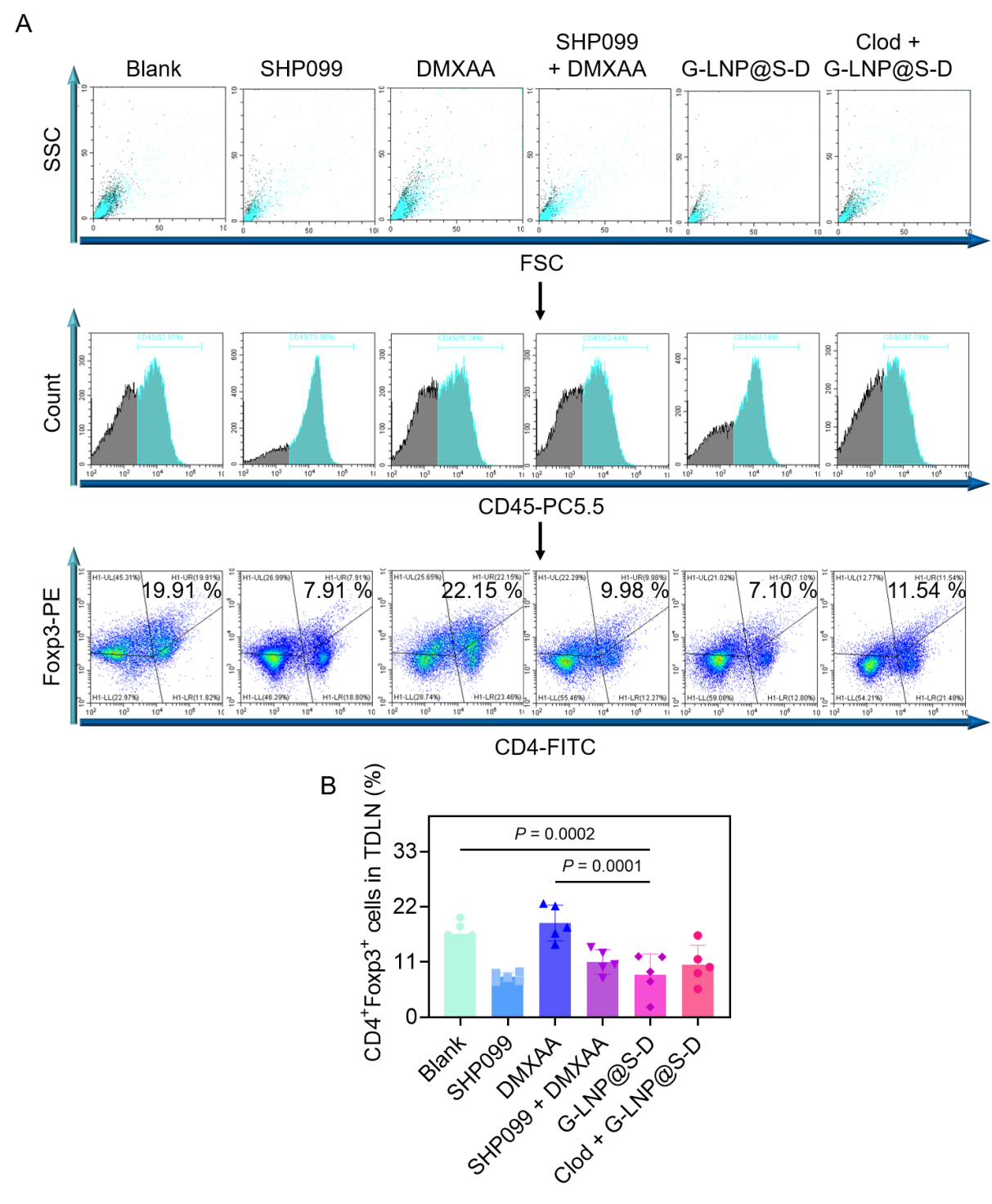


**Figure S18.** (A) Representative flow analysis chart and (B) corresponding quantitative analysis of CD4^+^Foxp3^+^ T regulatory (Treg) cells in TDLN after treatment with SHP099, DMXAA, SHP099 + DMXAA, G-LNP@S-D or Clod + G-LNP@S-D. P values were tested *via* a one-way ANOVA analysis.


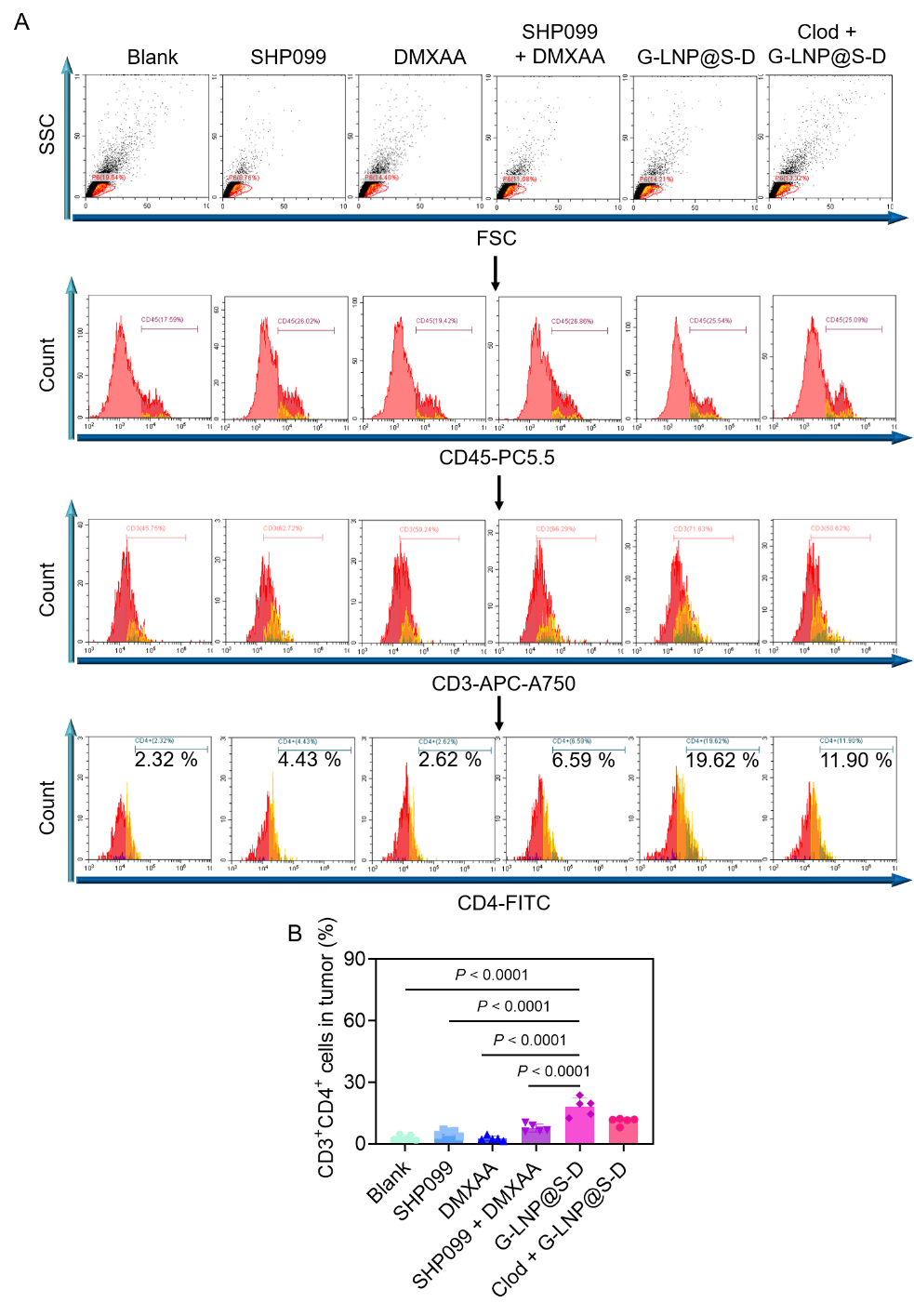


**Figure S19.** (A) Representative flow analysis chart and (B) corresponding quantitative analysis of CD3^+^CD4^+^ T lymphocytes in tumor after treatment with SHP099, DMXAA, SHP099 + DMXAA, G-LNP@S-D or Clod + G-LNP@S-D. P values were tested *via* a one-way ANOVA analysis.


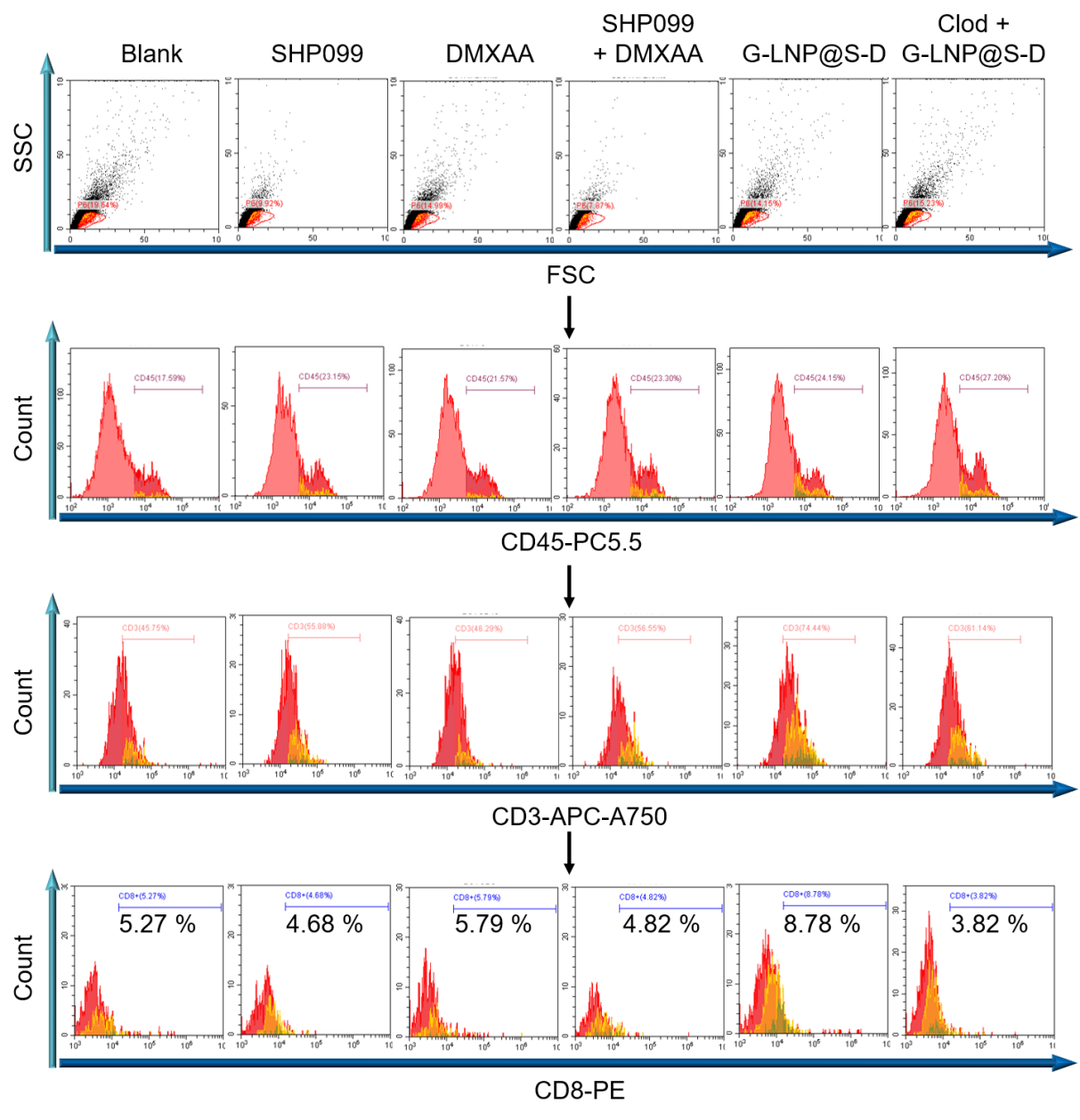


**Figure S20.** Representative flow analysis chart of CD3^+^CD8^+^ T lymphocytes in tumor after treatment with SHP099, DMXAA, SHP099 + DMXAA, G-LNP@S-D or Clod + G-LNP@S-D.


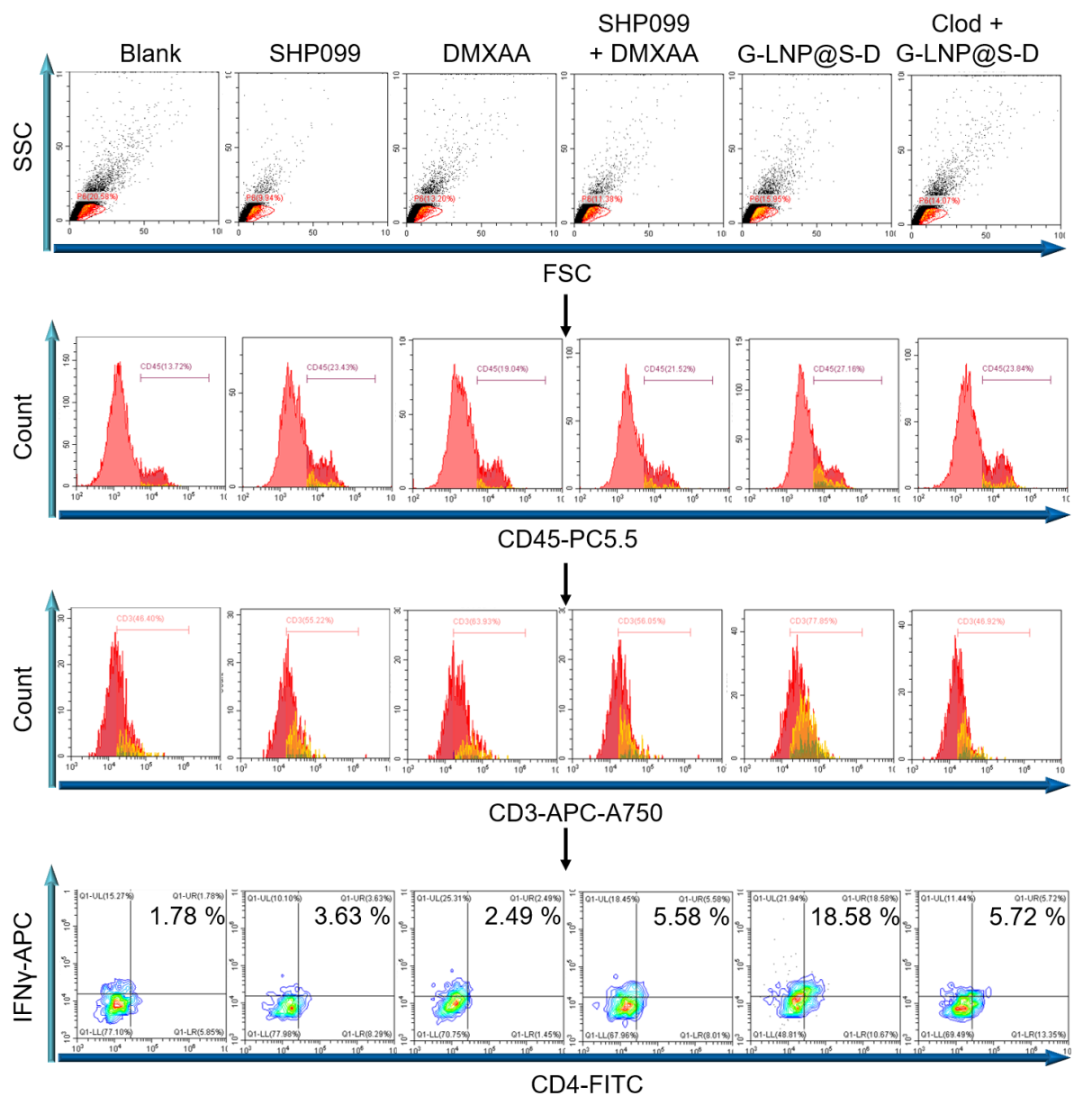


**Figure S21.** Representative flow analysis chart of CD4^+^IFNγ^+^ T lymphocytes in tumor after treatment with SHP099, DMXAA, SHP099 + DMXAA, G-LNP@S-D or Clod + G-LNP@S-D.


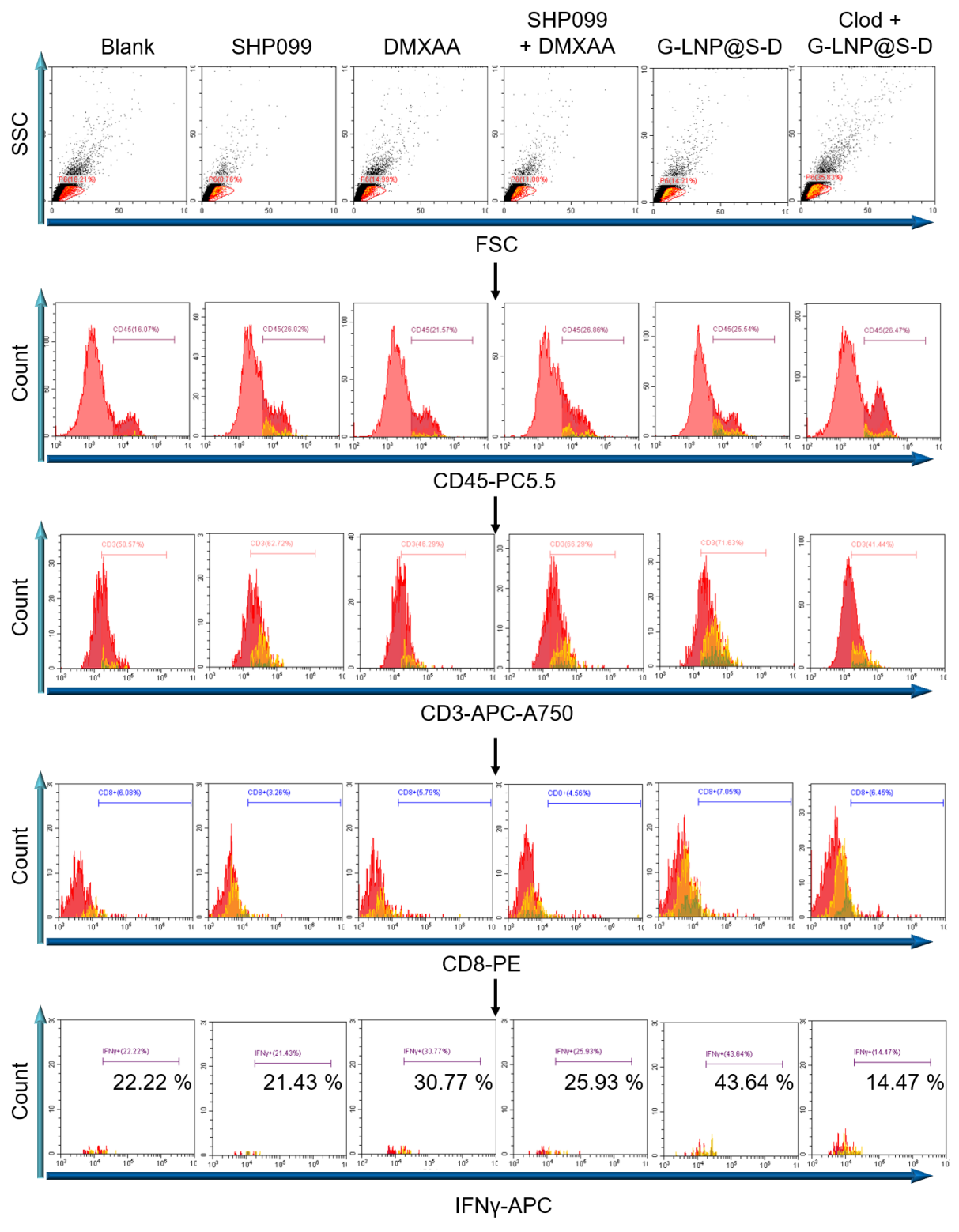


**Figure S22.** Representative flow analysis chart of CD8^+^IFNγ^+^ T lymphocytes in tumor after treatment with SHP099, DMXAA, SHP099 + DMXAA, G-LNP@S-D or Clod + G-LNP@S-D.


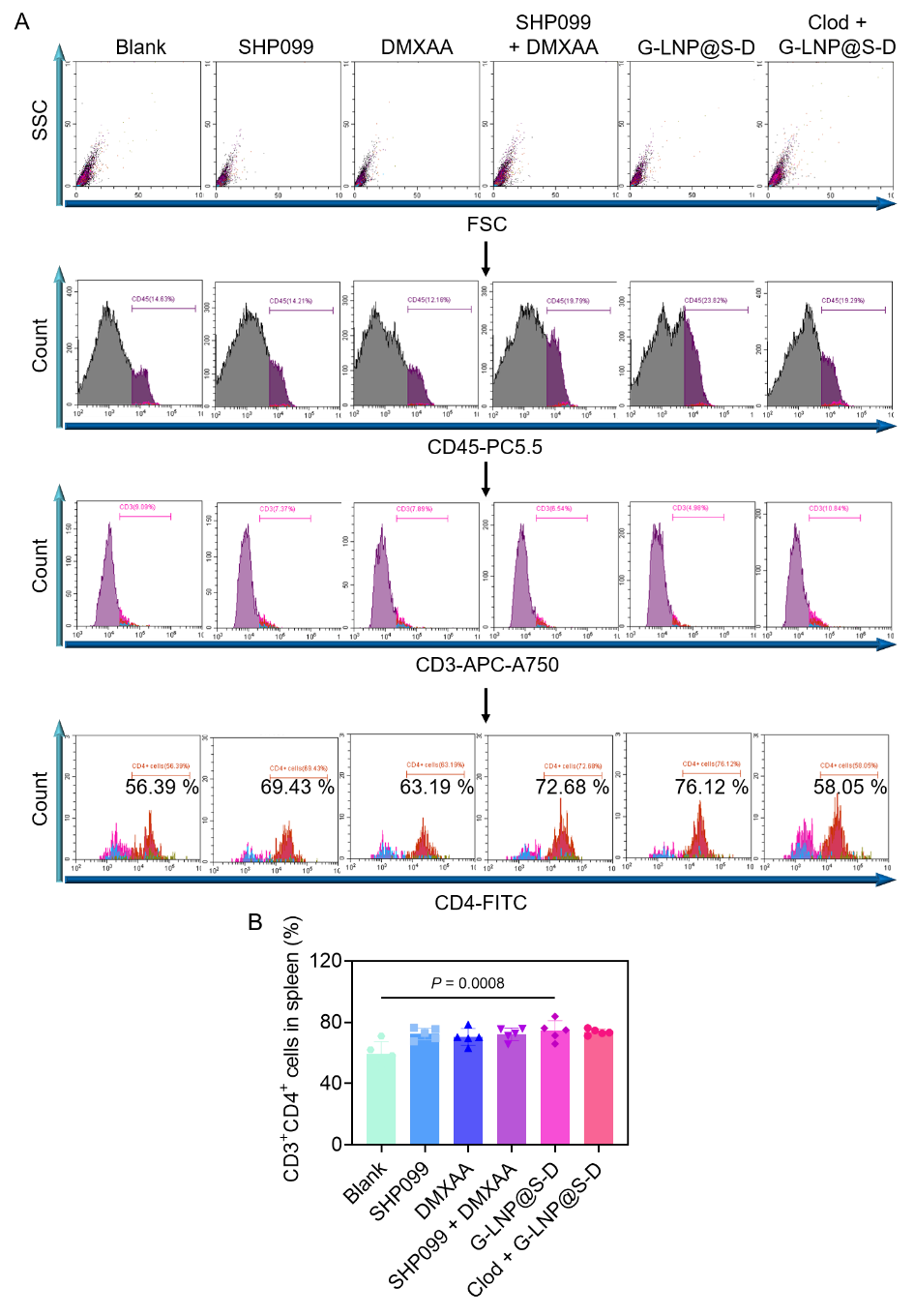


**Figure S23.** (A) Representative flow analysis chart and (B) corresponding quantitative analysis of CD3^+^CD4^+^ T lymphocytes in spleen after treatment with SHP099, DMXAA, SHP099 + DMXAA, G-LNP@S-D or Clod + G-LNP@S-D. P values were tested *via* a one-way ANOVA analysis.


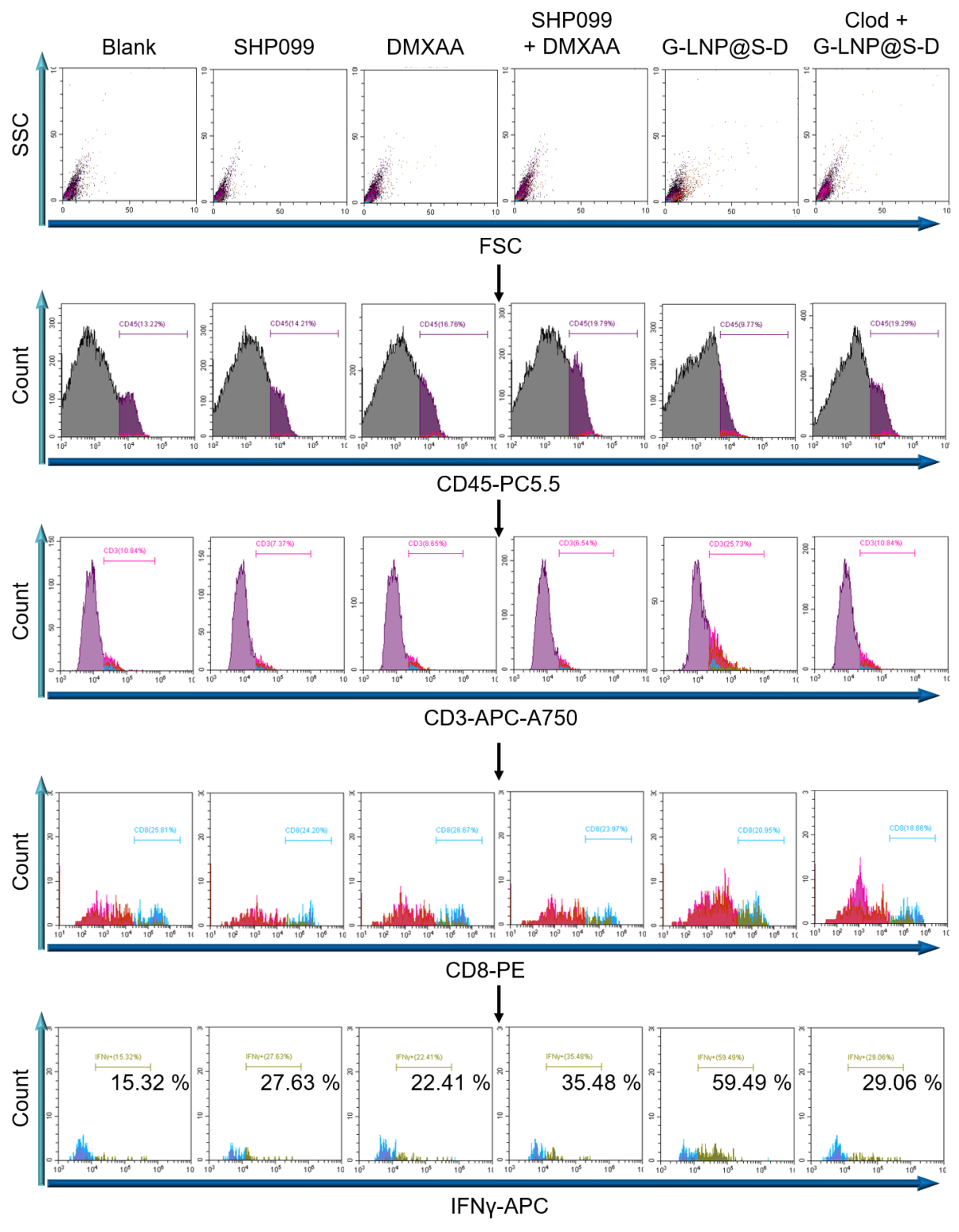


**Figure S24.** Representative flow analysis chart of CD8^+^IFNγ^+^ T lymphocytes in spleen after treatment with SHP099, DMXAA, SHP099 + DMXAA, G-LNP@S-D or Clod + G-LNP@S-D.


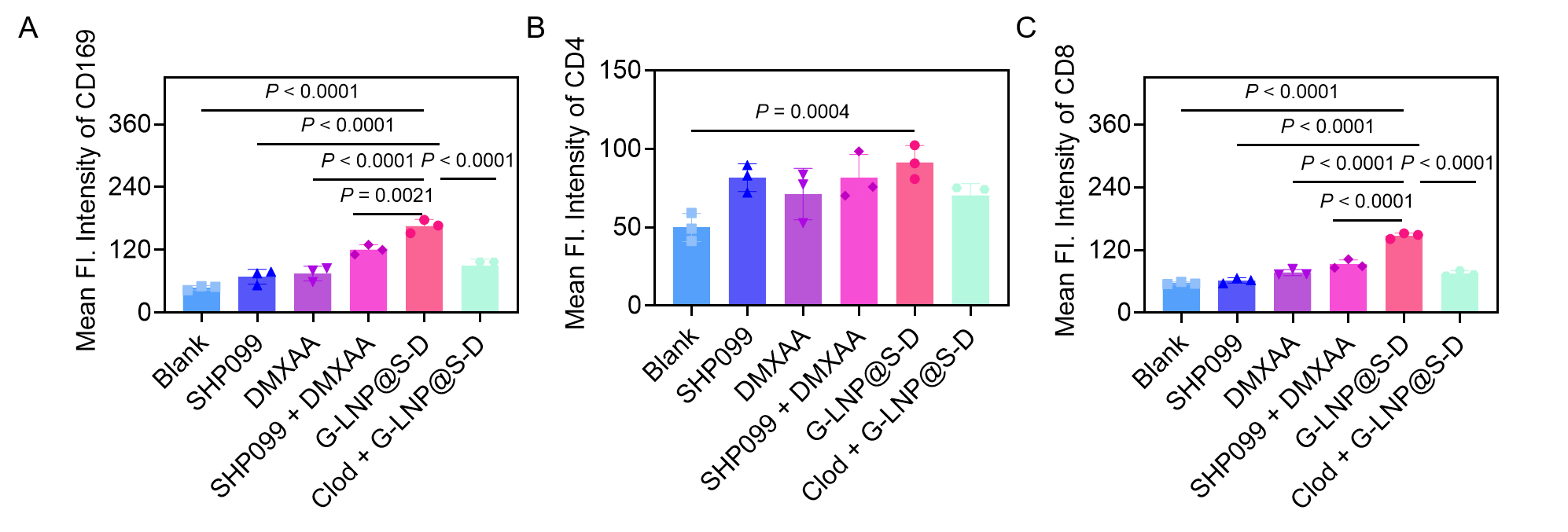


**Figure S25.** Corresponding quantitative immunofluorescence analysis of (A) CD169, (B) CD4 or (C) CD8 in TDLN after treatment with SHP099, DMXAA, SHP099 + DMXAA, G-LNP@S-D or Clod + G-LNP@S-D. P values were tested *via* a one-way ANOVA analysis.


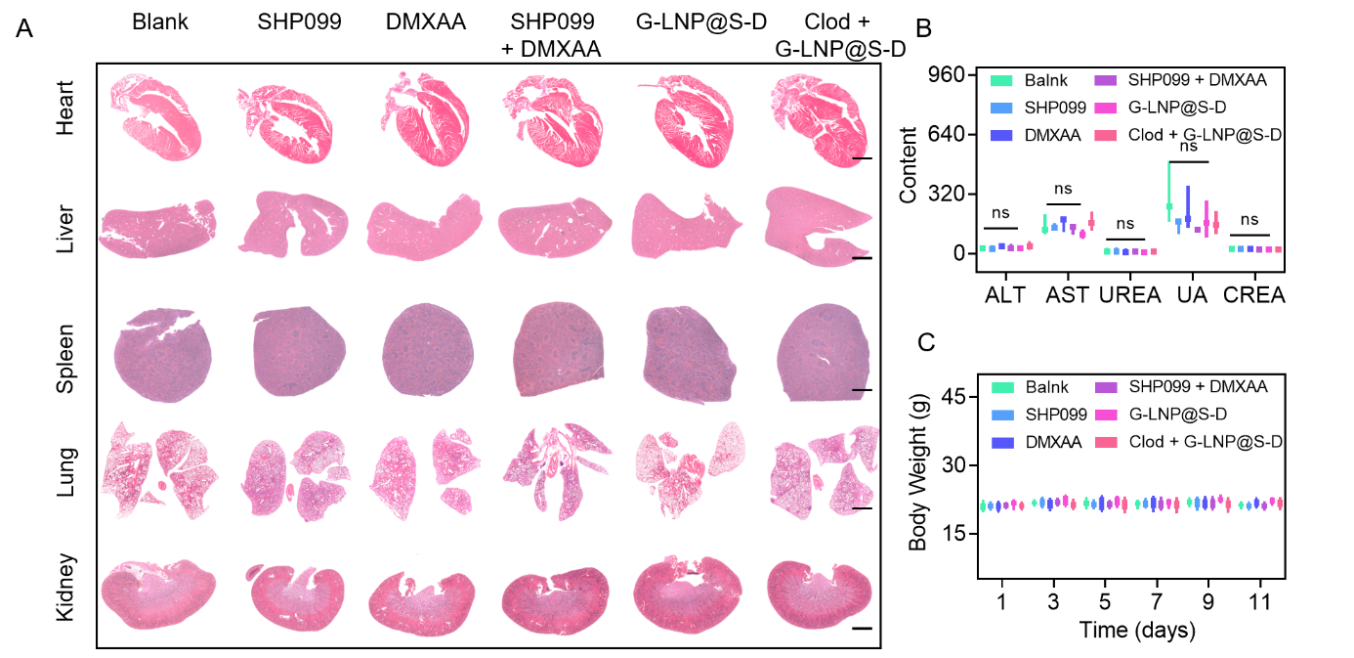


**Figure S26.** (A) H&E staining of the heart, liver, spleen, lung and kidney of the mice after treatment with SHP099, DMXAA, SHP099 + DMXAA, G-LNP@S-D or Clod + G-LNP@S-D on the 11th day. Scale bar: 200 µm. (B) The blood biochemical analysis (ALT, AST, UREA, UA, CREA) and (C) body weight variations of the mice after treatment with SHP099, DMXAA, SHP099 + DMXAA, G-LNP@S-D or Clod + G-LNP@S-D in 11 days. P values were tested *via* a one-way ANOVA analysis.
